# Verification of documentation plausibility in equine passports–drug documentation for geldings in comparison to self-reported veterinarian drug usage for equine castrations in Germany

**DOI:** 10.1371/journal.pone.0292969

**Published:** 2023-10-18

**Authors:** Shary Tamara Schneider, Rudi Isbrandt, Heidrun Gehlen, Nina Langkabel, Diana Meemken

**Affiliations:** 1 Institute of Food Safety and Food Hygiene, Working Group Meat Hygiene, School of Veterinary Medicine, Freie Universität Berlin, Berlin, Germany; 2 Clinic for Horses, School of Veterinary Medicine, Freie Universität Berlin, Berlin, Germany; University of Zambia, ZAMBIA

## Abstract

The ‘positive list for equines’ (Regulation (EC) No 1950/2006) was implemented in the European Union in 2006. The drugs listed are approved for use in slaughter equines under certain conditions, although those drugs are not approved for use in livestock and are not listed in Table 1 of the annex to Regulation (EU) No 37/2010. The usage of such drugs has to be documented in the equine passport and a withdrawal period of six months must be adhered to before the equine can be slaughtered for human consumption. Since the withdrawal period is long, correct documentation is particularly important. This study compared the results of two sub-studies. In sub-study 1, 116 veterinarians and nine equine clinics in Germany were surveyed about the methods and drugs used for castration of equine stallions. In sub-study 2, the documentational findings of 195 equine passports, belonging to 194 horses and one donkey, were analyzed. Regarding sub-study 1, the most commonly used method for castration was reported as ‘laid down’. Drug combinations entailing at least one drug from the ‘positive list’ were used by 86.7% (91/105) of veterinarians castrating horse stallions ‘laid down’ and by 64.3% (36/56) of veterinarians utilizing this method on donkey stallions. Regarding sub-study 2, drug documentation was verified in the passports of 4.6% (9/195) of all equines and in just 12.0% (3/25) of those belonging to slaughter equine geldings. Anesthetics from the ‘positive list’ were documented in 4.0% (1/25) of equine passports belonging to slaughter geldings. Because of the high discrepancy of the drug combinations used by veterinarians and the documentation actually found in equine passports, we conclude that drug administration is very seldom documented in equine passports in Germany. This could result in drug residues in equine meat and poses a potential risk for consumers.

## 1. Introduction

The equine passport was introduced in the European Union (EU) through Regulation (EC) No 504/2008 with the goal to implement an identification document for horses, donkeys, zebras, and hybrids thereof and to allow differentiation between slaughter and companion equines [1]. In 2015, the regulation was lifted through the implementation of the Implementing Regulation (EC) 2015/262, which in return was replaced by Implementing Regulation (EU) 2021/963 in July 2021 [2, 3]. To ensure adequate treatment options and access to medications of critical importance for slaughter equines, the so-called ‘positive list for equines’ (Regulation (EC) No 1950/2006), was compiled [4]. The ‘positive list’ is a directory with medications that are approved for use in slaughter equines under specific conditions in addition to medications approved for other livestock, as set in Table 1 of Regulation (EU) No 37/2010 [4, 5]. There are different regulations in place for the usage of medications on the ‘positive list’ than for other livestock-approved medications. For all medication listed in the ‘positive list’, the withdrawal period is six months, and each usage has to be documented in the equine passport. Another particularity regarding equines in the European legislation is the differentiation between slaughter equines (meat or other products from these animals can enter the human food chain) and companion equines (not allowed to be slaughtered for entry to the human food chain). For the treatment of companion equines, all medications that are allowed to be used in animals can be administered. However, if an equine is intended for slaughter to obtain meat for human consumption, certain medications are banned from use (usage of these medications is prohibited in livestock) or can only be used in compliance with particular regulations (medications on the ‘positive list’) [4, 5].

**Table 1:** Different drugs used for the castration of stallions while standing. Multiple answers could be given.

Drug	Horses (n = 27)		Donkeys (n = 13)		Classification regarding the usage in slaughter equines
	Frequency	Percentage %	Frequency	Percentage %	
Acepromazine	7	25.9	3	23.1	Permitted^a^
Butorphanol	14	51.9	6	46.2	Permitted^b^
Detomidine	13	48.1	5	38.5	Permitted^b^
Diazepam	1	3.7	1	7.7	Permitted^a^
Isoflurane	0	0.0	1	7.7	Permitted^b^
Ketamine	4	14.8	2	15.4	Permitted^b^
Levomethadone	10	37.0	4	30.8	Permitted^b^
Medetomidine	1	3.7	0	0.0	Prohibited^c^
Pentobarbital	1	3.7	1	7.7	Prohibited^c^
Xylazine	11	40.7	0	0.0	Permitted^b^

^a^ Drug listed in positive list Regulation (EC) No 1950/2006, withdrawal period six months [4]

^b^ Drug listed in Table 1 of Regulation (EU) No 37/2010 [5]

^c^ Drug not listed in Table 1 of Regulation (EU) No 37/2010 [5]

Because of the frequent changes in the regulations regarding the equine passport and amendments to the ‘positive list’, as well as the differentiation between slaughter and companion equines, the usage of medication in equines presents the veterinarians in Germany and the European Union with difficulties not faced in the treatment of other livestock or companion animals [1–4]. In a survey in 2013 in England, a total of 84% of veterinarians working with horses found the regulations regarding the English equine passport system difficult to understand, and 90% stated that the implementation of the equine passport did not fulfill its main purpose, food safety, because of non-compliance [6]. In our previous related study in Germany, a total of 83% of equine treating veterinarians perceived the overall documentation effort for slaughter equines as ‘large’ to ‘rather large’, and 64% perceived the regulations regarding the ‘positive list’ as ‘complicated’ to ‘rather complicated’ [7]. This lack of understanding of the regulations could contribute to the relatively high drug residue findings in equine meat in recent years in Germany (4.51% in 2018, 0.91% in 2019, and 1.87% in 2020) [8–10] and the EU (0.64% in 2020) [11]. In the same years (2018–2020), the percentages of positive poultry and pork samples were, respectively, 0.09%, 0.04%, 0.07% and 0.31%, 0.39%, and 0.26% [8–10]. This leads to the assumption that the documentation of medications in equine passports is likely insufficient and incomplete.

The objective of this study was to verify documentation plausibility in equine passports in Germany with regards to medications used in equine castrations. To achieve this, firstly, an overview of the commonly used medications for the different approaches to castrate horse and donkey stallions by veterinarians in Germany was generated. Secondly, how often medications were documented in the equine passport, especially anesthetics used for castration, was determined. Lastly, the correlation between usage of certain medications and frequency of documentation in the equine passport was also determined.

The study focused on castrations and the anesthetics used, and therefore, particularly on the anesthetics listed in the ‘positive list’ [4], since this procedure is easy to verify in the individual animals even after years.

## 2. Materials and methods

Data collection was performed via two different ways: 1) an online survey (active between 19^th^ October and 15^th^ December 2021) for different target groups and 2) through inspection of equine passports of individual animals at the clinic for horses of Freie Universität Berlin and in private stables (1^st^ June to 31^st^ December 2021).

The Central Ethics Committee of Freie Universität Berlin approved the approach of this study under ZEA-Nr.2022-013. The participants of the online survey gave consent to use their data through clicking of the survey start button. For the data collected from passport inspections, consent was given verbally by the equine owners that the data from their equine’s passport can be used. Since the study was designed for adults only and no personally identifying data was collected, the Central Ethics Committee waived the need for additional consent obtaining measures.

### 2.1 Online survey and questionnaires

The online survey was conducted among equine-attending veterinarians and equine clinics between 29^th^ October and 15^th^ December 2021 in Germany. The survey was created via Lime Survey Community Edition version 3.28.21 (LimeSurvey GmbH–Hamburg, Germany) and was hosted on a server of Freie Universität Berlin, Germany.

The questionnaires were developed in German by the authors of this study. A pre-test was performed between 19^th^ August to 15^th^ September 2021 with 13 veterinarians and three equine clinics, which resulted in seven completed questionnaires from veterinarians and two from the clinics. The feedback was used to improve the surveys´ usability, and the final questionnaires were edited accordingly. The results of the pre-test were not used for the final analysis.

The survey was accessed via an online link that was distributed through social media and mailing lists (S1 Table).

In total, 40 equine clinics were contacted directly via email. Of those, 35 were privately owned clinics, and each of the remaining five belonged to one of the five German universities for veterinary medicine. The clinics were selected to be geographically evenly distributed over Germany.

Since it is not possible to know how many veterinarians read the social media postings and invitation to the survey, the total number of contacted equine attending veterinarians cannot be estimated.

On the web site home page for the project, the research project was explained in short and data protection according to the current data protection laws in Germany was confirmed. It was stated that there are no affiliations to the official veterinary authorities of the German federal states, that the obtained data would not be given to law enforcement, and that the data would only be used confidentially and anonymously within the research project.

Once started, the survey had to be completed only in the given order, as answers for some questions were to clarify facts or were preconditions for the display of further questions. Questions could not be skipped forwards nor backwards, and therefore, answers could not be changed once given. This was implemented to minimize cheating on knowledge questions.

#### 2.1.1 Veterinarians´ questionnaire

The estimated maximum duration to complete the veterinarians´ questionnaire was 15 to 20 min. Depending on the answers given, a maximum of 66 questions were displayed (S1 File) and different or no follow-up questions were asked. For responses to be included in the study, a minimum of 17 questions had to be answered, and the veterinarians had to specify which method for castrating equine stallions was applied.

The questionnaire was designed with a mixture of free-text questions and selection-based questions (for example multiple or single choice).

The questionnaire covered the following topics: demographic questions, drug usage and documentation procedures, castration methods used, applicable regulations, and legislation regarding drug usage in equines and documentation procedure in Germany.

#### 2.1.2 Clinic questionnaire

The clinic questionnaire consisted of a maximum of 26 questions with an estimated duration to completion of 15 minutes (S2 File). Similarly to the veterinarians´ questionnaire, different or no follow-up questions were displayed depending on the answers to certain questions. For responses to be included in the study, all displayed questions had to be answered. The questionnaire consisted of a mixture of selection-based multiple or single choice and free-text questions. At the beginning, general questions about the castration procedure for horse and donkey stallions were asked, followed by more specific questions regarding the used medications.

### 2.2 Equine passport data

Collection of data from equine passports took place between 1^st^ June and 31^st^ December 2021 at the clinic for horses of the Freie Universität Berlin and in four horse retirement stables in three federal states (Brandenburg, Baden-Wuerttemberg, and Saxony-Anhalt). As for the university clinic for horses, once a week a trained research team member inspected the equine passports of the newly admitted equine patients in the clinic and collected the following information: type of equine (horse or donkey), sex, year of birth, castration status, year in which the castration was performed, documentation of the castration in the equine passport (yes or no), slaughter status (Is it a companion animal or destined for slaughter? Date of the documentation of the slaughter status? Is the documentation complete?), and if applicable, which administered drugs and medications were documented (S3 File). As for the horse retirement stables, data were collected only once using the same questionnaire as was used for inspecting the equine passports at the university clinic for horses.

The castration status was additionally verified through inspection of the equine, inquiry to the equine owner or equine keeper, or inspection of the patient file at the university clinic for horses of Freie Universität Berlin.

### 2.3 Data analysis

To be included, the veterinarians had to answer at least the question (F17 in the S1 File) ‘Do you castrate horse stallions: a) standing, b) lying down, c) both standing and lying down’. Due to the integration of incomplete datasets and that some questions were only displayed after giving a specific answer to a prior question, the reference values changed between the results obtained.

For the equine clinics, nine completely answered questionnaires were evaluated.

All data from equine passports were entered anonymously in an Excel table for further analysis. Descriptive data analysis was performed using and IBM SPSS version 28.0 for Windows (IBM® - Armonk, New York, USA). Frequency tables and figures were configured.

## 3. Results

In the following, the results of 119 veterinarians´ and nine clinics´ questionnaires and 195 equine passports are presented.

### 3.1 Results of the veterinarians’ questionnaire

Of the 119 veterinarians, 116 (97.5%) performed castrations on horse stallions and 69 (58.0%) on donkey stallions. Of the 116 veterinarians who performed castrations on horse stallions, the majority (75.9%, 88/116) did so while the horse was laid down, followed by ‘both standing and laid down’, while the least veterinarians did so only on a standing stallion (S1 Fig). Castration of donkey stallions was performed by 69 veterinarians. Similar to the horse stallions, most veterinarians (81.2%, 56/69) performed castrations on donkeys while laid down, followed by ‘both standing and laid down’ and ‘only standing’.

#### 3.1.1 Use of drugs for castration of stallions while standing

Of the 28 veterinarians that castrated a horse stallion while standing, 27 specified the medication they used (96.4%). Nine different drugs were named, of which two (22.2%) were drugs listed in the ‘positive list’ [4], as well as two (22.2%) drugs with generally prohibited usage in slaughter equines according to Regulation (EU) No 37/2010 (Table 1) [5]. The 13 veterinarians who specified the drugs they use for the castration of donkey stallions while standing named eight different drugs (Table 1). Out of these, two drugs (25.0%) were listed in the ‘positive list’ and one drug (12.5%), which is prohibited for usage in slaughter equines. Medetomidine and Xylazine were only reported for horses, while Isoflurane was only used for donkeys.

The named drugs for horse castrations were reportedly combined in 12 different variations (Table 2). Four (33.3%) of these combinations included at least one drug from the ‘positive list’, which were used by eight of 27 (29.6%) of the veterinarians. Two (16.7%) of the combinations included at least one drug that is prohibited for usage in slaughter equines. In total, these combinations were used by two of 27 (7.4%) of the veterinarians (Table 2).

**Table 2:** Drug or drug combination used for the castration of horse and donkey stallions while standing specified by veterinarians using this castration method.

Drug or drug combination	Horses (n = 27)		Donkeys (n = 13)		Classification regarding drug usage in slaughter equines
	Frequency	Percentage %	Frequency	Percentage %	
Acepromazine, Butorphanol, Detomidine	1	3.7	1	7.7	Permitted^a^
Acepromazine, Ketamine, Levomethadone	1	3.7	0	0.0	Permitted^a^
Acepromazine, Levomethadone, Xylazine	5	18.5	2	15.4	Permitted^a^
Butorphanol, Detomidine	11	40.7	3	23.1	Permitted^b^
Butorphanol, Detomidine, Pentobarbital	1	3.7	1	7.7	Prohibited^c^
Butorphanol, Xylazine	1	3.7	1	7.7	Permitted^b^
Diazepam, Ketamine, Xylazine	1	3.7	1	7.7	Permitted^a^
Ketamine, Levomethadone, Xylazine	1	3.7	0	0.0	Permitted^b^
Isoflurane	0	0.0	1	7.7	Permitted^b^
Ketamine, Xylazine	1	3.7	1	7.7	Permitted^b^
Levomethadone	1	3.7	0	0.0	Permitted^b^
Levomethadone, Xylazine	2	7.4	2	15.4	Permitted^b^
Medetomidine	1	3.7	0	0.0	Prohibited^c^

^a^ At least one drug listed in the positive list of Reg. (EC) No 1950/2006, withdrawal period six months [4]

^b^ All drugs listed in Table 1 of Reg. (EU) No 37/2010 [5]

^c^ At least one drug not listed in Table 1 of Reg. (EU) No 37/2010 [5]

Altogether, nine different drug combinations for the castration of a donkey stallion while standing were reported (Table 2). Three (33.3%) of these combinations contained at least one drug from the ‘positive list’. Four of 13 veterinarians used a drug combination with at least one drug from the ‘positive list’ (30.8%). One of the nine combinations contained a drug the usage of which is prohibited in slaughter equines (11.1%). This combination was used by one of the 13 veterinarians (7.7%) castrating donkeys (Table 2).

For horses and donkeys, the most commonly used drug combination was ‘Butorphanol, Detomidine’. This combination was reportedly used for horses by eleven of 27 veterinarians (40.7%) and for donkeys by three of 13 veterinarians (23.1%).

#### 3.1.2 Use of drugs for castration of stallions while laid down

Of the participating 116 veterinarians, who performed castrations on horse stallions, a total of 105 (90.5%) did so while they were laid down. As well as 59 (85.5%) of the 69 veterinarians, who castrate donkey stallions. For horse stallions, the usage of 14 different drugs was reported, as was the usage of 13 different drugs for donkey stallions (Table 3). In the case of four of 14 drugs used for horses, the drugs were listed in the ‘positive list’ (28.6%). One of the 14 mentioned drugs was prohibited in slaughter equines (7.1%). For donkeys, four of the 13 used drugs were from the ‘positive list’ (30.8%) (Table 3).

**Table 3:** Drugs used for the castration of stallions while laid down; multiple answers could be given.

Drug	Horses (n = 105)		Donkeys (n = 59)		Classification regarding drug usage in slaughter equines
	Frequency	Percentage %	Frequency	Percentage %	
Acepromazine	12	11.4	4	6.8	Permitted^a^
Butorphanol	24	22.9	18	30.5	Permitted^b^
Detomidine	21	20.0	18	30.5	Permitted^b^
Diazepam	69	65.7	36	61.0	Permitted^a^
Guaifenesin	23	21.9	10	16.9	Permitted^a^
Isoflurane	22	21.0	9	15.3	Permitted^b^
Ketamine	99	94.3	53	89.8	Permitted^b^
Nitrous oxide	1	1.0	1	1.7	Permitted^b^*
Levomethadone	7	6.7	4	6.8	Permitted^b^
Medetomidine	1	1.0	0	0	Prohibited^c^
Midazolam	1	1.0	0	0	Permitted^a^
Propofol	0	0	1	1.7	Permitted^a^
Romifidine	25	23.8	15	25.4	Permitted^b^
Thiopental	3	2.9	1	1.7	Permitted^b^
Xylazine	63	60.0	33	55.9	Permitted^b^

^a^ Drug listed in the positive list of Regulation (EC) No 1950/2006, withdrawal period six months [4]

^b^ Drug listed in Table 1 of Regulation (EU) No 37/2010 [5]

^c^ Drug not listed in Table 1 of Regulation (EU) No 37/2010 [5]

* Food additive in the EU, registered substance E942

The veterinarians castrating horse stallions while laid down used 14 drugs alone or in combination. In total, 61 different combinations were reported (S2 Table). Of these combinations, 49 contained at least one drug from the ‘positive list’ (80.3%). In total, 91/105 (86.7%) of the participating veterinarians used one of the 49 combinations. One combination included a drug that is prohibited from usage in slaughter equines (1.6%). This combination was used by one of the 105 veterinarians (1.0%). One combination was not classifiable (1.0%), since the answer was imprecise (‘Diazepam, Ketamine, Romifidine, Inhalation anesthesia’). The most commonly used drug combination contained the drugs Diazepam, Ketamine, and Xylazine, which were used by 14 of 105 of the veterinarians (13.3%).

The 13 different drugs used for donkey stallion castration while the animals were laid down were combined in 37 different combinations, of which 22 (59.5%) contained a drug from the ‘positive list’ (S3 Table). In total, 36 of 56 veterinarians (64.3%) used one of these 22 combinations. Two of the answers given could not be classified (5.4%), since the answers were imprecise.

### 3.2 Results of the equine clinics’ questionnaire

All nine surveyed equine clinics performed castrations while the equine was laid down and none while standing. In total, seven of the nine clinics reported they castrate horse and donkey stallions (77.8%), while the other two castrated only horse stallions (22.2%). All nine clinics stated that the used anesthetics did not vary depending on the slaughter status of the equine.

In total, the usage of 12 different anesthetics for the castration of horse stallions was reported in eight different combinations (Table 4). Drugs from the ‘positive list’ were present in seven of the eight combinations (87.5%) that were used by seven of the nine clinics (77.8%). For the castration of donkey stallions, the equine clinics reported the usage of eleven different anesthetics (Table 4). Drugs from the ‘positive list’ were present in seven of the eight combinations (87.5%), which were used by all of the seven clinics that castrated donkey stallions.

**Table 4:** Drug combinations used in equine clinics for the castration of horse and donkey stallions while laid down.

Drug combination	Horses (n = 9)		Donkeys (n = 7)		Classification regarding drug usage in slaughter equines
	Frequency	Percentage %	Frequency	Percentage %	
Acepromazine, Butorphanol, Detomedine, Diazepam, Isoflurane, Ketamine, Xylazine	1	11.1	1	14.3	Permitted^a^
Diazepam, Ketamine, Romifidine	1	11.1	1	14.3	Permitted^a^
Diazepam, Ketamine, Xylazine	2	22.2	2	28.6	Permitted^a^
Butorphanol, Diazepam, Isoflurane, Ketamine, Levomethadone, Xylazine	1	11.1	0	0	Permitted^a^
Butorphanol, Isoflurane, Ketamine, Romifidine, Thiopental	1	11.1	0	0	Permitted^b^
Fenpipramide hydrochloride, Guaifenesin, Isoflurane, Ketamine, Levomethadone, Xylazine	1	11.1	0	0	Permitted^a^
Fenpipramide hydrochloride, Isoflurane, Ketamine, Levomethadone, Xylazine	0	0	1	14.3	Permitted^a^
Diazepam, Isoflurane, Ketamine Romifidine, Xylazine	1	11.1	0	0	Permitted^a^
Butorphanol, Diazepam, Levomethadone, Ketamine, Xylazine	1	11.1	1	14.3	Permitted^a^
Levomethadone, Xylazine, or Butorphanol in combination with Diazepam, Ketamine, Isoflurane, and Xylazine continuous drip infusion	0	0	1	14.3	Permitted^a^

^a^ At least one drug listed in the positive list of Reg. (EC) No 1950/2006, withdrawal period six months [4]

^b^ All drugs listed in Table 1 of Reg. (EU) No 37/2010 [5]

Overall, eight of the nine equine clinics (88.9%) reported they deviated in less than 5% of the castrations from their respective standard medication. One of the nine clinics (11.1%) reported that they deviated in 25% to 50% from their standard medication. The reported reasons for a change in medication were poor response to Romifidine, increased need for drugs of the equine treated, complications, pain, drug intolerance, drug availability, preliminary malignant hyperthermia, and slaughter status.

In total, seven of the nine (77.8%) equine clinics stated that the treatment of anesthesia incidents in slaughter equines is more complicated than in companion equines, with two clinics (22.2%) stating that there are no approved drugs available.

### 3.3 Results of equine passport inspections

In total, the equine passports of 195 equines were inspected, which belonged to 194 (99.5%) horses and one female donkey (0.5%); of all passports, 54.9% were for males and 45.1% for females. Of the male equines, 98 of 107 (91.6%) were castrated. For 65 of 98 geldings (66.3%), the castration was not documented in the equine passport. On average, the equines were born in 2009, with the oldest horse born in 1990 and the youngest in 2021.

The appearance of the equine passports differed depending on the organization that issued the passport and the state or region where the equine was born.

All results are detailed in the S4 Table.

Overall, 47 of the 195 (24.1%) equines were intended for slaughter, 46 horses and one donkey. Of the companion equines, 44 of 148 (29.7%) were not documented correctly because of missing signature of the owner, missing signature of the veterinarian, or missing date, and therefore, these animals would have been slaughter equines under the previous applicable equine passport regulation (Implementing Regulation (EU) 2015/262 [4]) (S2 Fig).

Administered drugs were documented in nine of 195 (4.6%) equine passports (Table 5). These equine passports were for five companion equines and four slaughter equines. Overall, two of the administered drug documentations referred to the application of anesthetics from the ‘positive list’ [4] (once Diazepam in a slaughter equine and once Diazepam with Guaifenesin in a companion equine). One documentation was for the usage of Detomidine. The other documentations referred to application of anthelmintics or antibiotics.

**Table 5:** Frequency of documentational findings in equine passports regarding administered drugs depending on the sex of the equines and the slaughter status.

Slaughter status	Mares	Male equines		All sexes
		Geldings	Stallions	
Slaughter equines	1/20 (5.0%)	3/25 (12.0%)	0/2 (0.0%)	4/47 (8.5%)
Companion equines	1/68 (1.5%)	3/73 (4.1%)	1/7 (14.3%)	5/148 (3.4%)
Total	2/88 (2.3%)	6/98 (6.1%)	1/9 (11.1%)	9/195 (4.6%)

## 4. Discussion

According to our results the most commonly used castration method for horse and donkey stallions is with the animal laid down, as over three quarters (88/116) of independent working veterinarians use this method on horses and over 80% (56/69) on donkeys. However, both castration methods, laid down and standing, are in accordance with the guidelines for diligence during castration of the German Equine Veterinary Association [12]. The covered or semi-covered castration under general anesthesia (laid down) is deemed to have the lowest complication rate and healing time [12]. This may be one reason why castrating equine stallions while the animal is laid down is more frequently used by the surveyed veterinarians than castrating while standing. The method used should be chosen in consultation with the equine owner [12], as the resulting costs differ greatly [13].

Of the drug combinations used for castration while standing, one third included at least one drug from the ‘positive list’ (for horses 4 of 12 combinations and for donkeys 3 of 9 combinations), and these were used by approximately 30% of the participating veterinarians. The laid down method had a higher discrepancy in the percentage of drug combinations used between horses and donkeys. For horses, approximately 87% (91/105) of the veterinarians and 78% (7/9) of the clinics in the study used a combination containing at least one drug from the ‘positive list’, while for donkeys, this was the case for 64% (36/56) of the veterinarians and all clinics. Accordingly, we expected to find documentation of administered anesthetics from the ‘positive list’ in 30% to 87% of the equine passports belonging to gelding horses and in 30% to 100% of passports belonging to gelding donkeys, that are slaughter equines. Taking into consideration the distribution of the used methods, the percentage of drug documentation findings in equine passports should be closer to 87% and 100% for horse and donkey geldings, respectively, than to 30%. However, administered drugs were documented only in 12% (3/25) of equine passports belonging to slaughter equine geldings. Most notably, only 4% (1/25) of the equine passports belonging to slaughter equine geldings contained documentation of anesthetics from the ‘positive list’. Based on our numbers, we conclude that drug documentation for the castration of slaughter equines geldings is incorrect or missing in 26% to 96% of equine passports. Therefore, we assume that drug documentation in equine passports is in general often not conducted.

Incomplete drug usage documentation is not only a problematic practice regarding the equine population, as shown in a study among pig and cattle farms and pig- and cattle-treating veterinarians where incomplete records in 9.4% of cases for farms and 3.1% of veterinary practices were found [14]. While those numbers are high, they are noticeably lower than our estimates regarding incomplete drug usage documentation in equine passports. Incomplete drug usage documentation may be more prevalent among equine treating veterinarians than livestock treating veterinarians. A possible reason for this difference might be the more complex regulations for drug documentation for equines than for other livestock. For slaughter equines the regulations regarding medications approved for livestock (Regulation (EU) No 37/2010) apply, as well as the regulations regarding drug usage of the ‘positive list’ [4, 5]. Furthermore, the differentiation in companion and slaughter animals within the same species with distinct applicable regulations, is unique for equines [3]. Other species are either all classified as slaughter animals, for instance pigs, or as companion animals, e.g. cats.

The lack of documentation in equine passports could be the result of a lack of knowledge regarding the applicable drug documentation procedures, as over 50% of German equine treating veterinarians have only moderate to no knowledge of the regulation regarding the ‘positive list’ [7]. Although none of the observed equine passports lacked the basic information required [3], the general layout of the equine passports differed depending on the institution issuing the passport as well as between the different European countries. This might further complicate correct and thorough documentation for veterinarians, in particular in stressful clinical and practical settings when time for documentation is limited.

In total, three of the answers regarding the used drug documentations for equine castrations could not be classified since the answers were too vaguely formulated. The usage of ‘inhalation anesthetics’ was mentioned twice and ‘Alpha 2 agonists’ were mentioned once. Both drug groups include drugs that are approved for use in animals intended for human consumption, and therefore slaughter equines, as well as other drugs that are prohibited in these animals [5].

In over 65% (65/98) of the equine passports belonging to geldings, the castration was not documented. This is clearly problematic, because due to the castration, the appearance of the animal is altered, unequivocal identification by means of the equine passport is no longer possible. The geldings with incomplete documentation should, therefore, be excluded from entering the food chain. Fortunately, in the new model document for the equine passports a dedicated field for the documentation of the castration was implemented [3]. It would be interesting if future research could show a significant improvement of castration documentation. However, the EU member states are free to deviate from the new model document and implement their own version.

One of the most likely reasons for drug residues in meat might be failure to adhere to the drug withdrawal period [15]. For drugs from the ‘positive list’, the correct documentation of the withdrawal period of six months in equine passports is particularly important to avoid any loss of information. A lack of drug documentation in the equine passport clearly could result in a potential risk of drug residues for equine meat consumers. This risk is confirmed by the continuously higher drug and chemical residue findings in equine meat than in other meats in Germany [8–10]. In 2018, around 4.5% of tested equine meat samples were positive for these residues in Germany in contrast to approximately 0.3% of pork and 0.6% of beef samples [8]. In 2019, the discrepancy between positive tested samples was lower, but still high, with < 1% of equine meat samples tested positive in comparison to < 0.4% of pork and around 0.5% of beef samples [9]. In 2020, equine meat samples again more frequently contained drug residues than did pork or beef (nearly 1.9%, < 0.3% and < 0.8%, respectively) [10]. This trend was also noticeable at EU level, as in 2019 and 2020 > 1% of tested products derived from horses were positive for non-steroidal anti-inflammatory drugs in comparison to < 0.5% of bovine samples and < 0.05% of pig and poultry samples [11]. In human medicine, the documentation of used drugs is accepted as an essential part of medical care that reduces possible risks and increases therapeutical outcomes [16]. Thorough documentation of administered drugs could be similarly beneficial for individual equines, and by implication, a lack of correct documentation could adversely affect an individual equine’s welfare.

The participating veterinarians and the equine clinics often reported the use of combinations of four or more different drugs, even though with each additional drug used, the overall documentation effort increases. The practice of combining different drugs has proven to reduce the overall needed dosage, improve the effectiveness and reduce adverse effects of the drugs used for operations on a standing equine [17].

Since the equine passport inspections only included the passport of one donkey, the results cannot be representative of the German donkey population. The ratio of 194 horses to one donkey roughly matches the distribution of the equine population in Germany, with approximately 10,000 to 20,000 donkeys (Interessengemeinschaft der Esel- und Mulifreunde in Deutschland e.V., personal communication) to around 1.7 million horses [18, 19]. For both the veterinarian and clinic results, some selection bias has to be mentioned, since the study was online, and consequently, only veterinarians and clinics with internet access, which is broadly available in Germany, were included. For the sake of anonymity of the participants, the questionnaires did not include questions regarding personal information, such as ‘years of practice experience’. Therefore, it is unclear whether there is a correlation between ‘years of practice experience’ and the knowledge regarding drug documentation in equine passports.

Acknowledging the limitations of the study, we stand by our assumption that the lack of documentation in equine passports, and so the associated risks for equine meat consumer and the equine population can be generalized for Germany.

## 5. Conclusion

In conclusion, drug documentation in passports of equines intended for slaughter is seldom completed, leading to a potential health risk of drug residue consumption for equine meat consumers in Germany, as well as compromising the health of the individual equines. Further research is necessary to determine if missing and incomplete documentation castrations and administered drugs and in equine passports is a problem in the European Union. The implementation of Regulation (EU) 2021/963 is a step toward greater equine meat consumer safety [3]. It will be interesting to see if a positive impact thereof will be shown through future research. Additionally, awareness regarding the risks to humans and equines that is posed by any lack of drug documentation should be heightened among equine treating veterinarians.

## Supporting information

S1 Fig Methods used for the castration of equine stallions. (TIF)

S2 Fig Slaughter status in proportion to the sex of the equines (n = 195). (TIF)

S1 Table Survey distribution methods. (DOCX)

S2 Table Drug combination used for the castration of horse stallions while laid down (n = 105). (DOCX)

S3 Table Drug combinations used for the castration of donkey stallions while laid down (n = 59). (DOCX)

S4 Table Equine passport inspection results. (XLSX)

S1 File Veterinarians´ questionnaire. (PDF)

S2 File Clinics´ questionnaire. (PDF)

S3 File Equine passport data collection form. (PDF)

S4 File STROBE checklist. (DOCX)
