# Supplementary figures and images for: Verification of documentation plausibility in equine passports–drug documentation for geldings in comparison to self-reported veterinarian drug usage for equine castrations in Germany
Source: PLoS One. 2023 Oct 18;18(10):e0292969. doi: 10.1371/journal.pone.0292969 (PMC10584153; doi:10.1371/journal.pone.0292969)

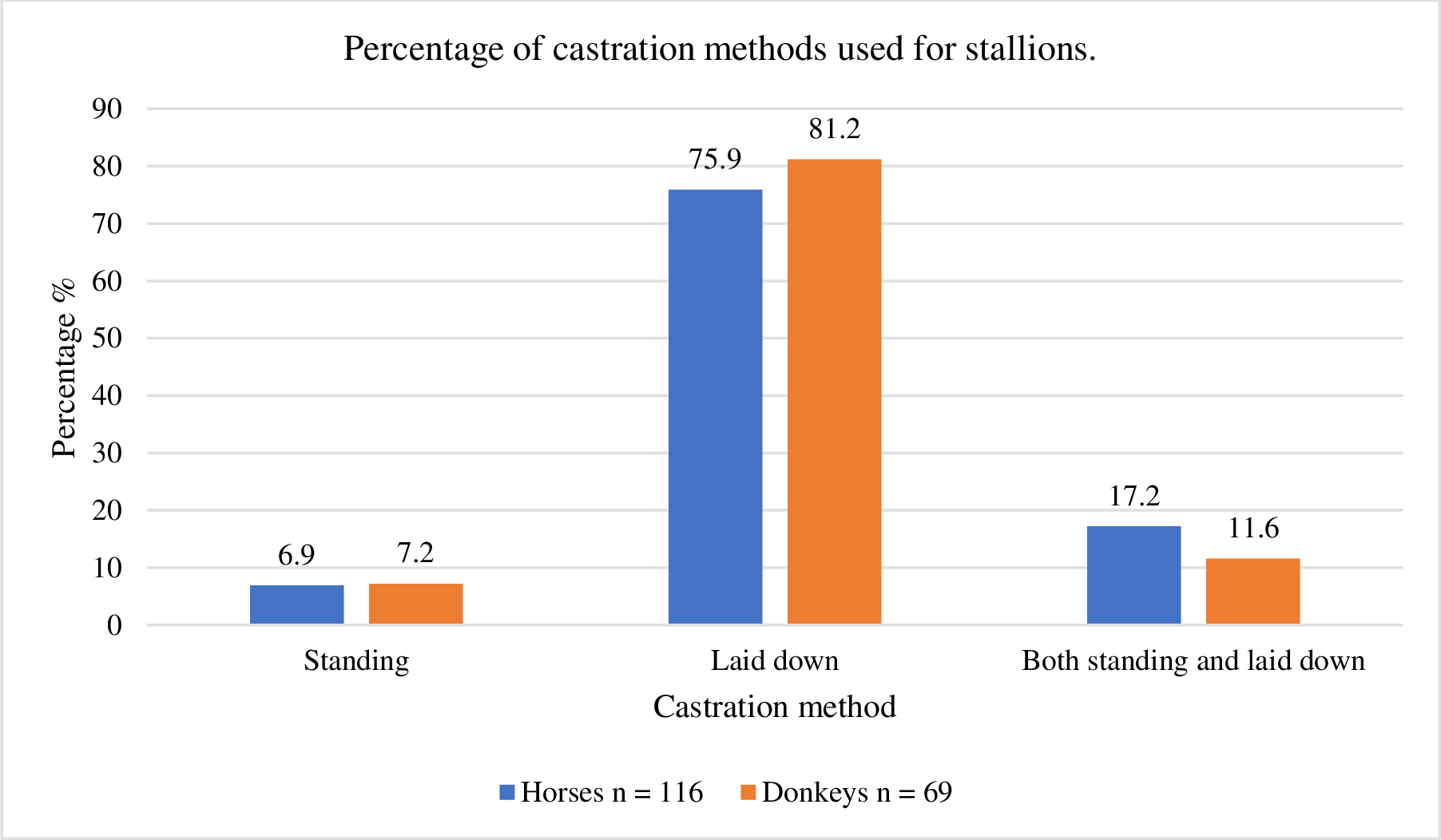

Supplement: S1 Fig — (TIF) [file pone.0292969.s001.tif]

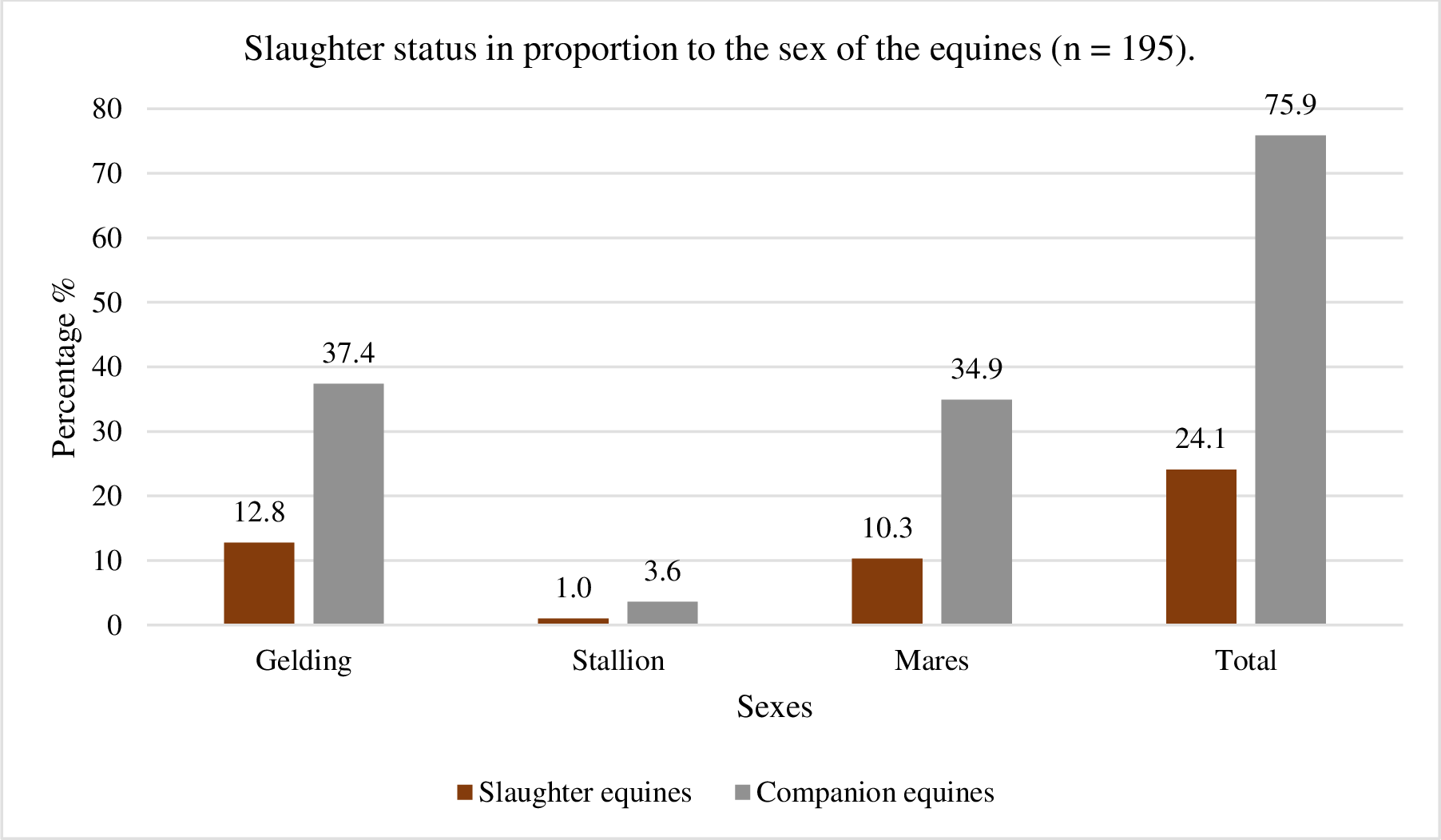

Supplement: S2 Fig — (TIF) [file pone.0292969.s002.tif]
