# Supplementary material for: Verification of documentation plausibility in equine passports–drug documentation for geldings in comparison to self-reported veterinarian drug usage for equine castrations in Germany
Source: PLoS One. 2023 Oct 18;18(10):e0292969. doi: 10.1371/journal.pone.0292969 (PMC10584153; doi:10.1371/journal.pone.0292969)
Supplement: S1 Table — (DOCX) [file pone.0292969.s003.docx]

**S1 Table: Survey distribution methods.**

| **Contacted** | **Contacting method** | **Distributor** |
| --- | --- | --- |
| Equine clinics | Direct email | Research team member |
| Veterinarians | Link posted to seven German veterinarian groups (total number of members at the time of posting: 11,243) | Research team member via Facebook |
| Veterinarians | Link posted to two private pages (approximate number of followers at the time of posting: 500) | Research team member via Instagram |
| Veterinarians | Published link on website | German Veterinary Association of the federal states Baden-Wuerttemberg |
| Veterinarians | Mailing lists | - ‘German Veterinary Society’ (Deutsche Veterinärmedizinische Gesellschaft, DVG) - ‘German Equine Veterinary Association’ (Gesellschaft für Pferdemedizin, GPM) - German Veterinary Association of the federal state Saxony - German Veterinary Association of the federal states Schleswig-Holstein - German Veterinary Association of the federal state Thuringia |
