# Supplementary material for: Verification of documentation plausibility in equine passports–drug documentation for geldings in comparison to self-reported veterinarian drug usage for equine castrations in Germany
Source: PLoS One. 2023 Oct 18;18(10):e0292969. doi: 10.1371/journal.pone.0292969 (PMC10584153; doi:10.1371/journal.pone.0292969)
Supplement: S2 Table — (DOCX) [file pone.0292969.s004.docx]

**S2 Table: Drugs or drug combinations used for the castration of horse stallions while laid down (n = 105).**

| **Drug or drug combination** | **Frequency** | **Percentage %** | **Classification regarding the drug usage in slaughter equines** |
| --- | --- | --- | --- |
| Acepromazine, Butorphanol, Detomidine, Ketamine | 1 | 1.0 | Permitted^a^ |
| Acepromazine, Butorphanol, Diazepam, Ketamine, Xylazine | 1 | 1.0 | Permitted^a^ |
| Acepromazine, Butorphanol, Ketamine, Xylazine | 1 | 1.0 | Permitted^a^ |
| Acepromazine, Detomidine, Isoflurane, Xylazine | 1 | 1.0 | Permitted^a^ |
| Acepromazine, Diazepam, Guaifenesin, Isoflurane, Ketamine, Romifidine | 1 | 1.0 | Permitted^a^ |
| Acepromazine, Diazepam, Ketamine, Levomethadon, Romifidine, Xylazine | 1 | 1.0 | Permitted^a^ |
| Acepromazine, Diazepam, Ketamine, Levomethadon, Xylazine | 1 | 1.0 | Permitted^a^ |
| Acepromazine, Guaifenesin, Ketamine, Romifidine, Xylazine | 1 | 1.0 | Permitted^a^ |
| Acepromazine, Guaifenesin, Levomethadone, Thiopental | 1 | 1.0 | Permitted^a^ |
| Acepromazin, Isofluran, Ketamine, Xylazine | 1 | 1.0 | Permitted^a^ |
| Acepromazine, Ketamine, Xylazine | 1 | 1.0 | Permitted^a^ |
| Acepromazine, Ketamine, Romifidine, Xylazine | 1 | 1.0 | Permitted^a^ |
| Butorphanol, Detomidine, Diazepam, Guaifenesin, Xylazine | 1 | 1.0 | Permitted^a^ |
| Butorphanol, Detomidine, Diazepam, Isoflurane, Ketamine | 2 | 1.9 | Permitted^a^ |
| Butorphanol, Detomidine, Diazepam, Isoflurane | 1 | 1.0 | Permitted^a^ |
| Butorphanol, Detomidine, Diazepam, Ketamine, Romifidine | 1 | 1.0 | Permitted^a^ |
| Butorphanol, Detomidine, Diazepam, Ketamine | 3 | 2.9 | Permitted^a^ |
| Butorphanol, Detomidine, Diazepam, Ketamine, Xylazine | 1 | 1.0 | Permitted^a^ |
| Butorphanol, Diazepam, Guaifenesin, Isoflurane, Ketamine, Xylazine | 1 | 1.0 | Permitted^a^ |
| Butorphanol, Diazepam, Guaifenesin, Ketamine, Xylazine | 1 | 1.0 | Permitted^a^ |
| Butorphanol, Diazepam, Isoflurane, Ketamine, Xylazine | 1 | 1.0 | Permitted^a^ |
| Butorphanol, Diazepam, Ketamine | 1 | 1.0 | Permitted^a^ |
| Butorphanol, Diazepam, Ketamine, Romifidine | 1 | 1.0 | Permitted^a^ |
| Butorphanol, Diazepam, Ketamine, Xylazine | 4 | 3.8 | Permitted^a^ |
| Butorphanol, Ketamine, Romifidine | 2 | 1.9 | Permitted^b^ |
| Butorphanol, Ketamine, Xylazine | 1 | 1.0 | Permitted^b^ |
| Detomidine, Diazepam, Isoflurane, Ketamine, Nitrous oxide | 1 | 1.0 | Permitted^a^* |
| Detomidine, Diazepam, Ketamine | 1 | 1.0 | Permitted^a^ |
| Detomidine, Diazepam, Ketamine, Levomethadone, Xyalzine | 1 | 1.0 | Permitted^a^ |
| Detomidine, Diazepam, Ketamine, Xyalzine | 1 | 1.0 | Permitted^a^ |
| Detomidine, Guaifenesin, Ketamine | 1 | 1.0 | Permitted^a^ |
| Detomidine, Guaifenesin, Thiopental | 1 | 1.0 | Permitted^a^ |
| Detomidine, Isoflurane, Ketamine, Xylazine | 1 | 1.0 | Permitted^b^ |
| Detomidine, Ketamine | 2 | 1.9 | Permitted^b^ |
| Detomidine, Ketamine, Xyalzine | 1 | 1.0 | Permitted^b^ |
| Diazepam, Guaifenesin, Ketamine, Levomethadone, Xylazine | 1 | 1.0 | Permitted^a^ |
| Diazepam, Guaifenesin, Ketamine, Romifidine | 2 | 1.9 | Permitted^a^ |
| Diazepam, Guaifenesin, Ketamine, Romifidine, Xylazine | 1 | 1.0 | Permitted^a^ |
| Diazepam, Guaifenesin, Ketamine, Thiopental, Xylazine | 1 | 1.0 | Permitted^a^ |
| Diazepam, Guaifenesin, Ketamine, Xylazine | 2 | 1.9 | Permitted^a^ |
| Diazepam, Isoflurane, Ketamine | 1 | 1.0 | Permitted^a^ |
| Diazepam, Isoflurane, Ketamine, Romifidine | 2 | 1.9 | Permitted^a^ |
| Diazepam, Isoflurane, Ketamine, Xylazine | 4 | 3.8 | Permitted^a^ |
| Diazepam, Ketamine | 4 | 3.8 | Permitted^a^ |
| Diazepam, Ketamine, Levomethadone, Xylazine | 1 | 1.0 | Permitted^a^ |
| Diazepam, Ketamine, Romifidine | 6 | 5.7 | Permitted^a^ |
| Diazepam, Ketamine, Romifidine, Xylazine | 1 | 1.0 | Permitted^a^ |
| Diazepam, Ketamine, Romifidine, “Inhalation anesthesia” | 1 | 1.0 | Not classifiable |
| Diazepam, Guaifenesin, Ketamine, Romifidine, Xylazine | 2 | 1.9 | Permitted^a^ |
| Diazepam, Ketamine, Xylazine | 14 | 13.3 | Permitted^a^ |
| Guaifenesin, Isoflurane, Ketamine, Xylazine | 2 | 1.9 | Permitted^a^ |
| Guaifenesin, Ketamine, Romifidine | 1 | 1.0 | Permitted^a^ |
| Guaifenesin, Ketamine, Xylazine | 5 | 4.8 | Permitted^a^ |
| Isoflurane | 1 | 1.0 | Permitted^b^ |
| Isoflurane, Ketamine, Romifidine | 1 | 1.0 | Permitted^b^ |
| Isoflurane, Ketamine, Xylazine | 1 | 1.0 | Permitted^b^ |
| Ketamine, Levomethadone, Xylazine | 1 | 1.0 | Permitted^b^ |
| Ketamine, Medetomidine, Xylazine | 1 | 1.0 | Prohibited^c^ |
| Ketamine, Midazolam, Xylazine | 1 | 1.0 | Permitted^a^ |
| Ketamine | 1 | 1.0 | Permitted^b^ |
| Ketamine, Xylazine | 4 | 3.8 | Permitted^b^ |

^a^ At least one drug listed in positive list Reg. (EU) No 122/2013, withdrawal period six months

^b^ All drugs listed in Table 1 Reg. (EU) No 37/2010

^c^ At least one drug not listed in Table 1 Reg. (EU) No 37/2010

*As food additive in the EU, registered substance E942
