# Supplementary material for: Verification of documentation plausibility in equine passports–drug documentation for geldings in comparison to self-reported veterinarian drug usage for equine castrations in Germany
Source: PLoS One. 2023 Oct 18;18(10):e0292969. doi: 10.1371/journal.pone.0292969 (PMC10584153; doi:10.1371/journal.pone.0292969)
Supplement: S3 Table — (DOCX) [file pone.0292969.s005.docx]

**S3 Table: Drug combinations used for the castration of donkey stallions while laid down (n = 59).**

| **Drug combination** | **Frequency** | **Percentage %** | **Classification regarding drug usage in slaughter equines** |
| --- | --- | --- | --- |
| Acepromazine, Diazepam, Guaifenesin, Isoflurane, Ketamine, Romifidine | 1 | 1.7 | Permitted^a^ |
| Acepromazine, Diazepam, Ketamine, Levomethadone, Romifidine, Xylazine | 1 | 1.7 | Permitted^a^ |
| Acepromazine, Ketamine, Xylazine | 1 | 1.7 | Permitted^a^ |
| Acepromazine, Xylazine, Detomidine, Isoflurane | 1 | 1.7 | Permitted^a^ |
| Butorphanol, Detomidine, Diazepam, Guaifenesin, Xylazine | 1 | 1.7 | Permitted^a^ |
| Butorphanol, Detomidine, Diazepam, Isoflurane | 1 | 1.7 | Permitted^a^ |
| Butorphanol, Detomidine, Diazepam, Isoflurane, Ketamine | 1 | 1.7 | Permitted^a^ |
| Butorphanol, Detomidine, Diazepam, Ketamin | 3 | 5.1 | Permitted^a^ |
| Butorphanol, Detomidine, Diazepam, Ketamine, Romifidine | 1 | 1.7 | Permitted^a^ |
| Butorphanol, Detomidine, Diazepam, Ketamine, Xylazine | 1 | 1.7 | Permitted^a^ |
| Butorphanol, Detomidine, Ketamine | 1 | 1.7 | Permitted^b^ |
| Butorphanol, Detomidine, Ketamine, Xylazine | 1 | 1.7 | Permitted^b^ |
| Butorphanol, Diazepam, Ketamine, Romifidine | 2 | 3.4 | Permitted^a^ |
| Butorphanol, Diazepam, Ketamine, Xylazin | 2 | 3.4 | Permitted^a^ |
| Butorphanol, Ketamine, Romifidine | 2 | 3.4 | Permitted^b^ |
| Butorphanol, Ketamine, Xylazine | 2 | 3.4 | Permitted^b^ |
| Detomidine, Diazepam, Ketamine | 1 | 1.7 | Permitted^a^ |
| Detomidine, Diazepam, Isoflurane, Ketamine, Nitrous oxide | 1 | 1.7 | Permitted^a*^ |
| Detomidine, Diazepam, Ketamine, Xylazine | 1 | 1.7 | Permitted^a^ |
| Detomidine, Guaifenesin, Thiopenthal | 1 | 1.7 | Permitted^b^ |
| Detomidine, Ketamine | 2 | 3.4 | Permitted^b^ |
| Detomidine, Ketamine, Xylazine | 1 | 1.7 | Permitted^b^ |
| Diazepam, Guaifenesin, Ketamine, Propofol, Xylazin, “Inhalation anesthesia” | 1 | 1.7 | Not classifiable |
| Diazepam, Guaifenesin, Ketamine, Romifidine, Xylazine | 1 | 1.7 | Permitted^a^ |
| Diazepam, Isoflurane, Ketamine, Xylazin | 2 | 3.4 | Permitted^a^ |
| Diazepam, Ketamine | 1 | 1.7 | Permitted^a^ |
| Diazepam, Ketamine, Alpha 2 agonists | 1 | 1.7 | Not classifiable |
| Diazepam, Ketamine, Levomethadone, Xylazine | 1 | 1.7 | Permitted^a^ |
| Diazepam, Ketamine, Romifidine | 6 | 10.2 | Permitted^a^ |
| Diazepam, Ketamine, Xylazine | 5 | 8.5 | Permitted^a^ |
| Diazepam, Xylazine | 1 | 1.7 | Permitted^a^ |
| Guaifenesin, Isoflurane, Ketamine, Xylazine | 1 | 1.7 | Permitted^b^ |
| Guaifenesin, Ketamine, Xylazine | 4 | 6.8 | Permitted^b^ |
| Isoflurane, Ketamine, Romifidine | 1 | 1.7 | Permitted^b^ |
| Ketamine, Levomethadone, Xylazine | 1 | 1.7 | Permitted^b^ |
| Ketamine, Xylazine | 3 | 5.1 | Permitted^b^ |
| Levomethadone, Xylazine | 1 | 1.7 | Permitted^b^ |

^a^ At least one drug listed in the positive list of Reg. (EU) No 122/2013, withdrawal period six months

^b^ All drugs listed in Table 1 of Reg. (EU) No 37/2010

*As food additive in the EU, registered substance E942
