# Supplementary material for: Verification of documentation plausibility in equine passports–drug documentation for geldings in comparison to self-reported veterinarian drug usage for equine castrations in Germany
Source: PLoS One. 2023 Oct 18;18(10):e0292969. doi: 10.1371/journal.pone.0292969 (PMC10584153; doi:10.1371/journal.pone.0292969)
Supplement: S1 File — (PDF) [file pone.0292969.s007.pdf]

S1 File – Questionnaire for veterinarians

Tierarzneimitteldokumentation bei Equiden - Fragebogen für Tierärzte / Tierärztinnen	Veterinary drug documentation for equines - Questionnaire for veterinarians
Vielen Dank für Ihr Interesse und Ihre Unterstützung für das Forschungsvorhaben „Arzneimittelanwendung und Dokumentation bei Equiden“.	Thank you for your interest and support for the research project "Drug use and documentation in equines".
Mein Name ist Shary Schneider, ich bin Tierärztin und schreibe eine Doktorarbeit an der Freien Universität Berlin am Fachbereich Veterinärmedizin, Institut für Lebensmittelsicherheit und –hygiene.	My name is Shary Schneider, I am a veterinarian, and I am writing a doctoral thesis at the Freie Universität Berlin at the School of Veterinary Medicine, Institute for Food Safety and Hygiene.
Im Rahmen meiner Doktorarbeit befrage ich Tierbesitzer*innen, Stallbetreiber*innen und Tierärzte/Tierärztinnen zu verschiedenen Punkten der Arzneimittelanwendung bei Pferden und Eseln.	As part of my doctoral thesis, I am interviewing animal owners, stable owners, and veterinarians about various aspects of the use of medications in horses and donkeys.
Ziel dieser Befragung ist es, den Stand der aktuell bei Equiden praktizierten Tiermedizin abzubilden. So sollen langfristig Ansatzpunkte zur Verbesserung der Arzneimittelsicherheit bei Pferden und Eseln gefunden werden.	The aim of this survey is to map the state of veterinary medicine currently practiced for equids. In this way, starting points for improving drug safety in horses and donkeys will be found in the long term.
Der nachfolgende Fragebogen für Tierärzte/Tierärztinnen umfasst Fragen zur Demografie, Behandlung und Anwendungen von Arzneimitteln, sowie Arzneimitteldokumentation.	The following questionnaire for veterinarians includes questions on demographics, treatment and use of medications, and medication documentation.
Die Dauer der Befragung beträgt ca. 15-20 Minuten.	The duration of the survey is approximately 15-20 minutes.
Sämtliche Daten werden nach den Richtlinien der DSGVO (Datenschutz-Grundverordnung) streng vertraulich behandelt. Eine personenbezogene Darstellung, sowie die Weitergabe an Dritte sind grundsätzlich ausgeschlossen. Dieses Forschungsprojekt steht in keinerlei Verbindung zur behördlichen Überwachung.	All data will be treated as strictly confidential according to the guidelines of the DSGVO (Data Protection Regulation). Any personal representation or passing any data on to third parties are principally excluded. This research project is in no way connected to official monitoring.
Vielen Dank, dass Sie sich Zeit nehmen, um an dieser Studie teilzunehmen!	Thank you for taking the time to participate in this study!
In dieser Umfrage sind 66 Fragen enthalten.	There are 66 questions in this survey.

Demographische Fragen / Demographic questions

F 1

**In welchem Bundesland befindet sich Ihre Praxis? ***<br>Bitte wählen Sie nur eine der folgenden Antworten aus:<br><br>○ Baden-Württemberg<br>○ Bayern<br>○ Berlin<br>○ Brandenburg<br>○ Bremen<br>○ Hamburg<br>○ Hessen<br>○ Mecklenburg-Vorpommern<br>○ Niedersachsen<br>○ Nordrhein-Westfalen<br>○ Rheinland-Pfalz<br>○ Saarland<br>○ Sachsen<br>○ Sachsen-Anhalt<br>○ Schleswig-Holstein<br>○ Thüringen	**In which federal state is your practice located? ***<br>Please select only one of the following answers:<br><br>○ Baden-Wuerttemberg<br>○ Bavaria<br>○ Berlin<br>○ Brandenburg<br>○ Bremen<br>○ Hamburg<br>○ Hessia<br>○ Mecklenburg-Western Pomerania<br>○ Lower Saxony<br>○ North Rhine-Westphalia<br>○ Rhineland-Palatinate<br>○ Saarland<br>○ Saxony<br>○ Saxony-Anhalt<br>○ Schleswig-Holstein<br>○ Thuringia

F 2

**Wie viele Tierärzte/Tierärztinnen arbeiten in Ihrer Praxis? ***<br><br>In dieses Feld dürfen nur Zahlen eingegeben werden.<br>Bitte geben Sie Ihre Antwort hier ein:	**How many veterinarians are employed in the veterinary practices you own or are employed in?***<br><br>Only numbers may be entered in this field.<br>Please enter your answer here:

F 3

**Welchen Umkreis umfasst das Einzugsgebiet Ihrer Praxis? ***<br><br>Bitte wählen Sie nur eine der folgenden Antworten aus:<br>○ <20km<br>○ bis 50km<br>○ bis 75km<br>○ bis 100km<br>○ >100km	**What is the catchment area of your practice? ***<br><br>Please select only one of the following answers:<br><br>○ <20km<br>○ up to 50km<br>○ up to 75km<br>○ up to 100km<br>○ >100km

F 4

**Welchen Anteil an Ihren Patienten stellen Equiden (Pferde und Esel) dar? ***<br><br>Bitte wählen Sie nur eine der folgenden Antworten aus:<br>o <10%<br>o 10 bis <25%<br>o 25 bis <50%<br>o 50 bis <75%<br>o 75 bis ≤100%	**What is the estimated percentage of equine patients (horses and donkeys) in relation to all treated patients in your practice? ***<br><br>Please select only one of the following responses:<br>o <10%<br>o 10 to <25%<br>o 25 to <50%<br>o 50 to <75%<br>o 75 to ≤100%

F 5

**Wie viel Prozent der von Ihnen behandelten Equiden sind Lebensmittel liefernde Tiere? ***<br><br>Bitte wählen Sie nur eine der folgenden Antworten aus:<br>o <10%<br>o 10 bis <25%<br>o 25 bis <50%<br>o 50 bis <75%<br>o 75 bis ≤100%<br>o Weiß ich nicht	**What percentage of your equine patients are livestock equines and destined for slaughter? ***<br><br>Please select only one of the following responses:<br>o <10%<br>o 10 to <25%<br>o 25 to <50%<br>o 50 to <75%<br>o 75 to ≤100%<br>o I do not know

F 6

**Wie groß ist der Anteil der Fahrpraxis? ***<br><br>Bitte wählen Sie nur eine der folgenden Antworten aus:<br>o <10%<br>o 10 bis <25%<br>o 25 bis <50%<br>o 50 bis <75%<br>o 75 bis ≤100%	**What is the share of mobile practice in total business? ***<br><br>Please select only one of the following responses:<br>o <10%<br>o 10 to <25%<br>o 25 to <50%<br>o 50 to <75%<br>o 75 to ≤100%

F 7

**Haben Sie die Möglichkeit, Equiden stationär aufzunehmen? ***<br><br>Bitte wählen Sie nur eine der folgenden Antworten aus:<br>o Ja<br>o Nein	**Do you have the means to treat equines as in patients? ***<br><br>Please select only one of the following responses:<br>o Yes<br>o No

F 8

**Haben Sie einen Operationssaal für Equiden? ***<br><br>Bitte wählen Sie nur eine der folgenden Antworten aus:<br>o Ja<br>o Nein	**Do you have an operating theater for equines? ***<br><br>Please select only one of the following answers:<br>o Yes<br>o No

F 9

**Arbeiten Sie auch als amtliche(r) Tierarzt/Tierärztin an einem Schlachthof / einer Metzgerei, in dem / in der Pferde oder Esel geschlachtet werden? ***<br><br>Bitte wählen Sie nur eine der folgenden Antworten aus:<br>o Ja<br>o Nein	**Do you also work as an official veterinarian in an abattoir or butchery where horses or donkeys are slaughtered? ***<br><br>Please select only one of the following answers:<br>o Yes<br>o No

F 10

**Haben Sie seit 2019 eine amtliche Schlachttieruntersuchung im Rahmen einer Notschlachtung bei Equiden durchgeführt? ***<br><br>Diese Frage wird nur angezeigt, wenn folgende Bedingungen erfüllt sind:<br>Antwort war 'Ja' bei Frage '9'.<br>Bitte wählen Sie nur eine der folgenden Antworten aus:<br>o Ja<br>o Nein	**Have you conducted an official ante-mortem inspection as part of an emergency equine slaughter since 2019? ***<br><br>This question is only displayed if the following conditions are met:<br>Answer was 'Yes' to question 9<br>Please select only one of the following answers:<br>o Yes<br>o No

Behandlung von Equiden / Specialized questions – treatment of equines

F 11

**Behandeln Sie Pferde / Eseln ohne Beisein des Besitzers / der Besitzerin? ***<br><br>Bitte wählen Sie die zutreffende Antwort aus:<br>o immer<br>o häufig<br>o gelegentlich<br>o selten<br>o nie	**Do you treat horses / donkeys without the owner being present? ***<br><br>Please select the applicable answer:<br>o always<br>o frequently<br>o occasionally<br>o rarely<br>o never

F 12

**Behandeln Sie Pferde / Esel ohne Beisein des Tierhalters /der Tierhalterin (Stallbetreibers/Stallbetreiberin)? ***<br><br>Bitte wählen Sie die zutreffende Antwort aus:<br><br>o immer<br>o häufig<br>o gelegentlich<br>o selten<br>o nie	**Do you treat horses / donkeys without the animal keeper (stable owner) being present? ***<br><br>Please select the applicable answer:<br>o always<br>o frequently<br>o occasionally<br>o rarely<br>o never

F 13

Wer gibt in Ihrer Praxis Wurmkuren ab? *	Who disperses deworming treatments in your practice? *
Bitte wählen Sie alle zutreffenden Antworten aus:<br>○ Der Tierarzt / die Tierärztin<br>○ TFA unter Aufsicht<br>○ TFA eigenständig<br>○ Praktikant*innen unter Aufsicht<br>○ Praktikant*innen eigenständig<br>○ Sonstiges: ☐<br><br>Fall Sie "Sonstiges" wählen, können Sie dies im Kommentarfeld erläutern.	Please select all that apply:<br>○ The veterinarian<br>○ Veterinary assistant under supervision<br>○ Veterinary assistant independently<br>○ Interns under supervision<br>○ Interns independently<br>○ Other: ☐<br><br>If you select "Other", you can explain in the comment field.

F 14

An wen geben Sie Wurmkuren ab? *	To whom do you dispense worming products? *
Bitte wählen Sie alle zutreffenden Antworten aus:<br>○ An Pferdebesitzer*in<br>○ An Stallbetreiber*in<br>○ An Angestellte des betreuten Stalles<br>○ Ich hinterlege die Wurmkuren an einem bestimmten Ort, z.B. in der Stallgasse<br>○ Sonstiges: ☐<br><br>Fall Sie "Sonstiges" wählen, können Sie dies im Kommentarfeld erläutern.	Please select all that apply:<br>○ To horse owner<br>○ To the stable owner<br>○ To employees of the stable<br>○ I leave the deworming products in a specific place, e.g., in the stable aisle.<br>○ Other: ☐<br><br>If you select "Other", you can explain this in the comment field.

F 15

Unter welchen Bedingungen geben Sie Wurmkuren ab? *	Under what conditions do you give deworming treatments? *
Bitte wählen Sie alle zutreffenden Antworten aus:<br>○ Nach klinischer Untersuchung<br>○ Nach medizinischer Beratung/Aufklärung<br>○ Nur an persönlich bekannte/betreute Tiere/Bestände<br>○ An jeden<br>○ Sonstiges: ☐<br><br>Fall Sie "Sonstiges" wählen, können Sie dies im Kommentarfeld erläutern.	Please select all that apply:<br>○ After clinical examination<br>○ After medical consultation/education<br>○ Only to personally known/cared for animals/stock.<br>○ To anyone<br>○ Other: ☐<br><br>If you select "Other", you may explain in the comment field.

Standardmedikation / Spezialized questions – standard medication

F 16

Kastrieren Sie Pferdehengste? *	Do you castrate horse stallions? *
Bitte wählen Sie nur eine der folgenden Antworten aus:<br>o Ja<br>o Nein	Please select only one of the following answers:<br>o Yes<br>o No

F 17

Kastrieren Sie Pferdehengste: *	Do you castrate horse stallions: *
Diese Frage wird nur angezeigt, wenn folgende Bedingungen erfüllt sind: Antwort war 'Ja' bei Frage '16'.<br>Bitte wählen Sie nur eine der folgenden Antworten aus:<br>o Stehend<br>o Abgelegt<br>o Sowohl stehend, als auch abgelegt	This question will only be displayed if the following conditions are met: Answer was 'Yes' to question 16.<br>Please select only one of the following answers:<br>o Standing<br>o Lying down<br>o Both standing and lying down

F 18

Welche Standardmedikation verwenden Sie zur Sedierung / Narkose während der Kastration eines Hengstes? *	What standard medication do you use for sedation / anesthesia during castration of a stallion? *
Diese Frage wird nur angezeigt, wenn folgende Bedingungen erfüllt sind:<br>Antwort war 'Stehend' oder 'Abgelegt' bei Frage '17'.<br><br>Bitte geben Sie Ihre Antwort hier ein:<br>[ ]	This question will only be displayed if the following conditions are met:<br>Answer was 'Standing' or 'Lying down' for question 17.<br><br>Please enter your answer here:<br>[ ]

F 19

Welche Standardmedikation verwenden Sie zur Sedierung / Narkose während der Kastration eines Hengstes bei: *	What standard medication do you use for sedation / anesthesia during castration of a stallion for: *
Diese Frage wird nur angezeigt, wenn folgende Bedingungen erfüllt sind:<br>Antwort war 'Sowohl stehend, als auch abgelegt' bei Frage '17'.<br>*Stehend:*<br>[ ]<br>*Abgelegt:*<br>[ ]	This question will only be displayed if the following conditions are met:<br>Answer was 'Both standing and lying down' at question 17.<br>*Standing:*<br>[ ]<br>*Lying down:*<br>[ ]

F 20

**Wenden Sie bei Schlachtpferden bei der Kastration eine andere Art der Sedierung / Narkose im Vergleich zu Nicht-Schlachtpferden an? ***<br><br>Diese Frage wird nur angezeigt, wenn folgende Bedingungen erfüllt sind:<br>Antwort war 'Ja' bei Frage '16'.<br>Bitte wählen Sie nur eine der folgenden Antworten aus:<br>○ Ja<br>○ Nein	**Do you use a different type of sedation / anesthesia for castration in slaughter horses compared to companion horses? ***<br><br>This question is only displayed if the following conditions are met:<br>Answer was 'Yes' to question 16.<br>Please select only one of the following answers:<br>○ Yes<br>○ No

F 21

**In welchen Punkten unterscheidet sich die von Ihnen angewendete Sedierung / Narkose beim Schlachtpferd im Vergleich zum nicht-Schlachtpferd? ***<br><br>Diese Frage wird nur angezeigt, wenn folgende Bedingungen erfüllt sind:<br>Antwort war 'Ja' bei Frage '20'.<br>Bitte geben Sie Ihre Antwort hier ein:	**In what ways does the sedation / anesthesia you use differ in the slaughter horse compared to the companion horse? ***<br><br>This question is only displayed if the following conditions are met:<br>Answer was 'Yes' to question 20.<br>Please enter your answer here:

F 22

**Wie dokumentieren Sie die angewendete Sedierung / Narkose bei Schlachtpferden? ***<br><br>Diese Frage wird nur angezeigt, wenn folgende Bedingungen erfüllt sind:<br>Antwort war 'Ja' bei Frage '16'.<br>Bitte wählen Sie alle zutreffenden Antworten aus:<br>○ In der Patientenakte<br>○ Mit einem Anwendungs- und Abgabebeleg<br>○ Im Equidenpass<br>○ Sonstiges:<br><br>Falls Sie "Sonstiges" wählen, können Sie dies im Kommentarfeld erläutern.	**How do you document sedation / anesthesia used on slaughter horses? ***<br><br>This question is only displayed if the following conditions are met:<br>Answer was 'Yes' to question 16.<br>Please select all that apply:<br>○ In the patient record<br>○ With a drug application and dispersion form<br>○ In the equine passport<br>○ Other:<br><br>If you select "Other", you may explain in the comment field.

F 23

**Wie häufig weichen Sie bei Sedierung / Narkose zur Kastration eines Pferdes von der Standardmedikation ab? ***<br><br>Diese Frage wird nur angezeigt, wenn folgende Bedingungen erfüllt sind:<br>Antwort war 'Ja' bei Frage '16'.<br>Bitte wählen Sie nur eine der folgenden Antworten aus:<br>o <5%<br>o 5% bis <10%<br>o 10% bis <25%<br>o 25% bis <50%	**When sedating / anesthetizing a horse for castration, how often do you deviate from standard medication? ***<br><br>This question is only displayed if the following conditions are met:<br>Answer was 'Yes' to question 16.<br>Please select only one of the following answers:<br>o <5%<br>o 5% to <10%<br>o 10% to <25%<br>o 25% to <50%

F 24

**Kastrieren Sie Eselhengste? ***<br><br>Bitte wählen Sie nur eine der folgenden Antworten aus:<br>o Ja<br>o Nein	**Do you castrate donkey stallions? ***<br><br>Please select only one of the following answers:<br>o Yes<br>o No

F 25

**Kastrieren Sie Eselhengste: ***<br><br>Diese Frage wird nur angezeigt, wenn folgende Bedingungen erfüllt sind:<br>Antwort war 'Ja' bei Frage '24'.<br>Bitte wählen Sie nur eine der folgenden Antworten aus:<br>o Stehend<br>o Abgelegt<br>o Sowohl stehend, als auch abgelegt	**Do you castrate donkey stallions: ***<br><br>This question will only be displayed if the following conditions are met:<br>Answer was 'Yes' to question 24.<br>Please select only one of the following answers:<br>o Standing<br>o Lying down<br>o Both standing and down

F 26

**Welche Standardmedikation verwenden Sie zur Sedierung / Narkose während der Kastration eines Eselhengstes? ***<br><br>Diese Frage wird nur angezeigt, wenn folgende Bedingungen erfüllt sind:<br>Antwort war 'Stehend' oder 'Abgelegt' bei Frage '25'.<br>Bitte geben Sie Ihre Antwort hier ein:	**What standard medication do you use for sedation / anesthesia during castration of a donkey stallion? ***<br><br>This question will only be displayed if the following conditions are met:<br>Answer was 'Standing' or 'Lying down' for question 25.<br>Please enter your answer here:

F 27

**Welche Standardmedikation verwenden Sie zur Sedierung / Narkose während der Kastration eines Eselhengstes bei: ***<br><br>Diese Frage wird nur angezeigt, wenn folgende Bedingungen erfüllt sind:<br>Antwort war 'Sowohl stehend, als auch abgelegt' bei Frage '25'.<br>*Stehend:*<br>[ ]<br>*Abgelegt:*<br>[ ]	**What standard medication do you use for sedation / anesthesia during castration of a donkey stallion for: ***<br><br>This question will only be displayed if the following conditions are met:<br>Answer was 'Both standing and lying down' at question 25.<br>*Standing:*<br>[ ]<br>*Lying down*<br>[ ]

F 28

**Wenden Sie bei Schlachteseln bei der Kastration eine andere Art der Sedierung / Narkose im Vergleich zu nicht-Schlachteseln an? ***<br><br>Diese Frage wird nur angezeigt, wenn folgende Bedingungen erfüllt sind:<br>Antwort war 'Ja' bei Frage '24'.<br>Bitte wählen Sie nur eine der folgenden Antworten aus:<br>o Ja<br>o Nein	**Do you use a different type of sedation / anesthesia for slaughter donkeys during castration compared to non-slaughter donkeys? ***<br><br>This question is only displayed if the following conditions are met:<br>Answer was 'Yes' to question 24.<br>Please select only one of the following answers:<br>o Yes<br>o No

F 29

**In welchen Punkten unterscheidet sich die von Ihnen angewendete Sedierung / Narkose beim Schlachtesel im Vergleich zum nicht-Schlachtesel? ***<br><br>Diese Frage wird nur angezeigt, wenn folgende Bedingungen erfüllt sind:<br>Antwort war 'Ja' bei Frage '28'.<br>Bitte geben Sie Ihre Antwort hier ein:<br>[ ]	**In what ways does the sedation / anesthesia you use differ for the slaughter donkey compared to the companion donkey? ***<br><br>This question is only displayed if the following conditions are met:<br>Answer was 'Yes' to question 28.<br>Please enter your answer here:<br>[ ]

F 30

**Wie dokumentieren Sie die angewendete Sedierung / Narkose bei Schlachteseln? ***<br><br>Diese Frage wird nur angezeigt, wenn folgende Bedingungen erfüllt sind:<br>Antwort war 'Ja' bei Frage '24'.<br>Bitte wählen Sie alle zutreffenden Antworten aus:<br>○ In der Patientenakte<br>○ Mit einem Anwendungs- und Abgabebeleg<br>○ Im Equidenpass<br>○ Sonstiges:<br><br>Falls Sie "Sonstiges" wählen, können Sie dies im Kommentarfeld erläutern.	**How do you document sedation / anesthesia used for slaughter donkeys? ***<br><br>This question is only displayed if the following conditions are met:<br>Answer was 'Yes' to question 24.<br>Please select all that apply:<br>○ In the patient record<br>○ With a drug application and dispersion form<br>○ In the equine passport<br>○ Other:<br><br>If you select "Other", you may explain in the comment field.

F 31

**Wie häufig weichen Sie bei Sedierung / Narkose zur Kastration eines Esels von der Standardmedikation ab? ***<br><br>Diese Frage wird nur angezeigt, wenn folgende Bedingungen erfüllt sind:<br>Antwort war 'Ja' bei Frage '24'<br>Bitte wählen Sie nur eine der folgenden Antworten aus:<br>○ <5%<br>○ 5% bis <10%<br>○ 10% bis <25%<br>○ 25% bis <50%	**How often do you deviate from standard medication when sedating/anesthetizing to castrate a donkey? ***<br><br>This question is only displayed if the following conditions are met:<br>Answer was 'Yes' to question 24.<br>Please select only one of the following answers:<br>○ <5%<br>○ 5% to <10<br>○ 10% to <25<br>○ 25% to <50%

Anwendung von Tierarzneimitteln / Specialized questions – veterinary drug usage

F 32

**Welche NSAIDs werden bei Ihnen am häufigsten für Equiden verschrieben / abgegeben / angewandt? ***<br><br>Bitte geben Sie Ihre Antwort hier ein:<br>Bitte nennen Sie maximal 3 NSAIDs	**What NSAID(s) do you most frequently use/disperse / prescribe? ***<br><br>Please enter your answer here:<br>Please name a maximum of 3 NSAIDs

F 33

**Gibt es bei den am häufigsten verschriebenen / angewandten NSAIDs Unterschiede zwischen Schlachtequiden und Nicht-Schlachtequiden? ***<br><br>Bitte wählen Sie nur eine der folgenden Antworten aus:<br>○ Ja<br>○ Nein	**Are there differences in the most commonly prescribed/used NSAIDs between slaughter equines and companion equines? ***<br><br>Please select only one of the following responses:<br>○ Yes<br>○ No

F 34

**Wie häufig im Monat verschreiben oder wenden Sie Phenylbutazon bei Pferden/Eseln durchschnittlich an? ***<br><br>Bitte geben Sie Ihre Antwort hier ein:	**How often do you prescribe or use phenylbutazone in a horse/donkey on average in one month? ***<br><br>Please enter your answer here:

F 35

**Was machen Sie, wenn es sich bei dem mit Phenylbutazon zu behandelnden Tier um ein Schlachtpferd handelt? ***<br><br>Bitte wählen Sie nur eine der folgenden Antworten aus:<br>o Nichts<br>o Eintrag in den Equidenpass<br>o Umtragung des Schlachtstatus im Equidenpass<br>o Wahl eines anderen Medikaments<br>o Wahl eines anderen Medikaments plus Eintragung in Equidenpass oder Ausstellung eines AuA-Beleges<br>o Sonstiges:<br><br>Falls Sie "Sonstiges" wählen, können Sie dies im Kommentarfeld erläutern.	**How would you proceed if an equine that is scheduled to receive phenylbutazone is an equine destined for slaughter? ***<br><br>Please select only one of the following answers:<br>o Do nothing<br>o Document the usage of phenylbutazone in the equine passport.<br>o Change the status of the equine destined for slaughter to not allowed to be slaughtered meaning the horse is a companion animal. Use a different drug<br>o Use a different drug and document this usage in equine passport or in an ‘AuA-Beleg’.<br>o Other:<br><br>If you select "Other", you can explain this in the comment field.

F 36

**Empfehlen / verkaufen Sie CBD (Canabidiol, Stoff der Hanfpflanze)-haltige Futterergänzungsmittel? ***<br><br>Bitte wählen Sie nur eine der folgenden Antworten aus:<br>o Ja<br>o Nein	**Do you recommend / sell CBD (canabidiol, substance of the hemp plant)-containing feed supplements? ***<br><br>Please select only one of the following answers:<br>o Yes<br>o No

F 37

**Was machen Sie, wenn es sich bei dem mit Canabidiol zu behandelnden Tier um ein Schlachtpferd handelt? ***<br><br>Diese Frage wird nur angezeigt, wenn folgende Bedingungen erfüllt sind:<br>Antwort war 'Ja' bei Frage '36'.<br>Bitte wählen Sie nur eine der folgenden Antworten aus:<br>o Nichts<br>o Eintrag in den Equidenpass<br>o Umtragung des Schlachtstatus im Equidenpass<br>o Wahl eines anderen Medikaments<br>o Wahl eines anderen Medikaments plus Eintragung in Equidenpass oder Ausstellung eines AuA-Beleges<br>o Sonstiges: [ ]<br><br>Falls Sie "Sonstiges" wählen, können Sie dies im Kommentarfeld erläutern.	**What do you do if the animal to be treated with canabidiol is a slaughter horse? ***<br><br>This question is only displayed if the following conditions are met:<br>Answer was 'Yes' to question 36.<br>Please select only one of the following answers:<br>o Nothing<br>o Make an entry in the equine passport<br>o Transfer the slaughter status to companion status in the equine passport<br>o Choose another medication<br>o Choose another medication plus make an entry in the equine passport or issue a drug application and dispersion form<br>o Other: [ ]<br><br>If you choose "Other", you can explain this in the comment field.

F 38

**Wie häufig leiten Sie in etwa ein/e bakteriologische Untersuchung / Antibiogramm vor einer antibiotischen Behandlung ein? ***<br><br>Bitte wählen Sie nur eine der folgenden Antworten aus:<br>o Vor jeder Antibiotikagabe<br>o Vor 75% bis 100% der Antibiotikagaben<br>o Vor 50% bis <75% der Antibiotikagaben<br>o Vor 25% bis <50% der Antibiotikagaben<br>o Vor 10% bis <25% der Antibiotikagaben<br>o Vor <10% der Antibiotikagaben<br>o Nie<br>o Sonstiges: [ ]<br>Falls Sie "Sonstiges" wählen, können Sie dies im Kommentarfeld erläutern.	**Approximately how often do you initiate a bacteriological examination/antibiogram prior to antibiotic treatment? ***<br><br>Please select only one of the following responses:<br>o Before every antibiotic administration<br>o Before 75% to 100% of antibiotic administrations<br>o Before 50% to <75% of antibiotic administrations<br>o Before 25% to <50% of antibiotic administrations<br>o Before 10% to <25% of antibiotic administrations<br>o Before <10% of antibiotic administrations<br>o Never<br>o Other: [ ]<br><br>If you select "Other", you can explain in the comment field.

F 39

Deutsch	English
**Aus welchen Gründen leiten Sie eine bakteriologische Untersuchung ein? ***<br><br>Diese Frage wird nur angezeigt, wenn folgende Bedingungen erfüllt sind:<br>Antwort war 'Sonstiges' oder 'Vor <10% der Antibiotikagaben' oder 'Vor 10% bis <25% der Antibiotikagaben' oder 'Vor 25% bis <50% der Antibiotikagaben' oder 'Vor 50% bis <75% der Antibiotikagaben' oder 'Vor 75% bis 100% der Antibiotikagaben' oder 'Vor jeder Antibiotikagabe' bei Frage '38'<br><br>Bitte wählen Sie alle zutreffenden Antworten aus:<br>o Bei ausbleibendem Behandlungserfolg<br>o Bei Antibiotikaeinsatz<br>o Bei Einsatz von Reserveantibiotika<br>o Bei unklaren Infektion<br>o Bei Risikopatienten<br>o Sonstiges: ______<br><br>Falls Sie "Sonstiges" wählen, können Sie dies im Kommentarfeld erläutern.	**For what reasons do you initiate a bacteriological examination? ***<br><br>This question is only displayed if the following conditions are met:<br>Answer was 'Other' or 'Before <10% of antibiotic administrations' or 'Before 10% to <25% of antibiotic administrations' or 'Before 25% to <50% of antibiotic administrations' or 'Before 50% to <75% of antibiotic administrations' or 'Before 75% to 100% of antibiotic administrations' or 'Before each antibiotic administration' for question '38'.<br><br>Please select all that apply:<br>o If treatment is not successful<br>o When antibiotics are used<br>o If reserve antibiotics are used<br>o In case of unclear infection<br>o In the case of risk patients*<br>o Other: ______<br><br>If you select "Other", you can explain in the comment field.<br>* Patients who have prior existing health conditions or are likely to have adverse reactions to medications

F 40

Deutsch	English
**Wann wenden Sie Reserveantibiotika** an? ***<br><br>Bitte wählen Sie alle zutreffenden Antworten aus:<br>o Nach Antibiogramm<br>o Je nach Anamnese<br>o Wenn andere Antibiotika sich als nicht wirksam erwiesen haben<br>o Nach Erfahrungswerten<br>o Auf Wunsch des Besitzers<br>o Sonstiges: ______<br><br>**Cephalosporine der 3. und 4. Generation, sowie Fluorchinolone<br>Falls Sie "Sonstiges" wählen, können Sie dies im Kommentarfeld erläutern.	**When do you use antibiotics of critical importance**? ***<br><br>Please select all that apply:<br>o According to antibiogram<br>o According to medical history<br>o When other antibiotics have not proven effective<br>o According to experience<br>o At the request of the owner<br>o Other: ______<br><br>**Cephalosporins of the 3rd and 4th generation, as well as fluoroquinolones.<br>If you select "Other", you can explain in the comments field.

F 41

Kennen Sie die Vorgaben der TÄHAV-Novelle von 2018 zum Einsatz von Reserveantibiotika (Cephalosporine der 3. und 4. Generation, sowie Fluorchinolone) bei Pferden? *	How well do you know the specifications of the national regulation regarding veterinary drug usage and distribution of medication (TÄHAV, 2018) regarding the use of antibiotics of critical importance (3rd and 4th generation cephalosporins, and fluoroquinolones) in horses? *
Bitte wählen Sie die zutreffende Antwort aus:<br>o Sehr gut<br>o Gut<br>o Mäßig<br>o Schlecht<br>o Gar nicht	Please select the answer that applies:<br>o Very well<br>o Well<br>o Moderately<br>o Poorly<br>o Not at all

F 42

Wie empfinden Sie die Komplexität der Regelungen zur Antibiogrammpflicht? *	How do you perceive the complexity of the antibiogram regulations? *
Bitte wählen Sie die zutreffende Antwort aus:<br>o Einfach<br>o Eher einfach<br>o Weder noch<br>o Eher kompliziert<br>o kompliziert	Please select the answer that applies:<br>o Simple<br>o Rather simple<br>o Neither simple nor complicated<br>o Rather complicated<br>o Complicated

Dokumentationsvorschriften / Spezialized questions – documentation

F 43

Kennen Sie die Dokumentationsvorschriften für Schlachtequiden? *	How well do you know the regulations regarding the documentation for equines destined for slaughter? *
Bitte wählen Sie die zutreffende Antwort aus:<br>o Sehr gut<br>o Gut<br>o Mäßig<br>o Schlecht<br>o Gar nicht	Please select the answer that applies:<br>o Very well<br>o Well<br>o Moderately<br>o Poorly<br>o Not at all

F 44

Wie häufig sehen Sie die Equidenpässe ein? *	When do you inspect the equine passport before treating an equine? *
Bitte wählen Sie nur eine der folgenden Antworten aus:<br>o Nie<br>o Einmal bei Aufnahme des Patienten<br>o Vor größeren Eingriffen<br>o Bei jeder Behandlung<br>o Sonstiges: ______<br><br>Falls Sie "Sonstiges" wählen, können Sie dies im Kommentarfeld erläutern.	Please select only one of the following answers:<br>o Never<br>o Once when the patient is admitted<br>o Before major surgery<br>o During every treatment<br>o Other: ______<br><br>If you select "Other", you can explain this in the comment field.

F 45

**Haben Sie Pferde oder Esel als Patienten, die vor dem 30.06.2009 geboren wurden? ***<br><br>Bitte wählen Sie nur eine der folgenden Antworten aus:<br>o Ja<br>o Vielleicht<br>o Nein	**Do you have horses or donkeys as patients born before 06/30/2009? ***<br><br>Please select only one of the following responses:<br>o Yes<br>o Maybe<br>o No

F 46

**Haben Sie schon einmal ein Fohlen behandelt, das noch keinen Pass hatte? ***<br><br>Bitte wählen Sie nur eine der folgenden Antworten aus:<br>o Ja, häufig<br>o Ja, gelegentlich<br>o Ja, selten<br>o Nein, noch nie	**Have you ever treated a foal that did not have a passport? ***<br><br>Please select only one of the following answers:<br>o Yes, frequently<br>o Yes, occasionally<br>o Yes, rarely<br>o No, never

F 47

**Wie häufig kommt der Fall eines behandlungsbedürftigen Fohlens ohne Equidenpass in Prozent zu allen anderen Pferdepatienten vor? ***<br><br>Diese Frage wird nur angezeigt, wenn folgende Bedingungen erfüllt sind:<br>Antwort war 'Ja, häufig' oder 'Ja, gelegentlich' oder 'Ja, selten' bei Frage '46'<br>Bitte geben Sie Ihre Antwort hier ein:	**What is the frequency in percent of a case of a foal in need of treatment without an equine passport in proportion to all other equine patients? ***<br><br>This question is only displayed if the following conditions are met:<br>Answer was 'Yes, frequently' or 'Yes, occasionally' or 'Yes, rarely' for question '46'.<br>Please enter your answer here:

F 48

**Wie gehen Sie vor, wenn der Pferdepass nicht vorliegt? ***<br><br>Diese Frage wird nur angezeigt, wenn folgende Bedingungen erfüllt sind:<br>Antwort war 'Ja, häufig' oder 'Ja, gelegentlich' oder 'Ja, selten' bei Frage '46'<br>Bitte wählen Sie nur eine der folgenden Antworten aus:<br>o Ich behandele das Fohlen<br>o Ich behandele das Fohlen nur mit Medikamenten, die für Lebensmittel liefernde Tiere zugelassen sind<br>o Ich behandele das Fohlen nur mit Medikamenten, die für Lebensmittel liefernde Tiere zugelassen sind und stelle einen AuA-Beleg aus	**What do you do if the equine passport is not available? ***<br><br>This question is only displayed if the following conditions are met:<br>Answer was 'Yes, frequently' or 'Yes, occasionally' or 'Yes, rarely' for question '46'.<br>Please select only one of the following answers:<br>o I treat the foal<br>o I treat the foal only with medications approved for food-producing animals<br>o I treat the foal only with medicines approved for food-producing animals and issue an AuA voucher.<br>o I treat the foal only with medications that are approved for food-producing animals or with medications from the positive list and enter them later in the horse passport.

○ Ich behandele das Fohlen nur mit Medikamenten, die für Lebensmittel liefernde Tiere zugelassen sind, bzw. mit Medikamenten der Positivliste und trage es später in den Pferdepass ein<br>○ Ich behandele das Fohlen und trage es als Lebensmittel lieferndes Tier aus, sobald es einen Pferdepass hat<br>○ Ich beantrage einen „Notfall-Transponder“ und behandele es mit für Lebensmittel liefernde Pferde zugelassenen Medikamenten<br>○ Ich kann das Fohlen nicht behandeln und schläfere es ein<br>○ Sonstiges: [ ]<br><br>Falls Sie "Sonstiges" wählen, können Sie dies im Kommentarfeld erläutern.	○ I treat the foal and register it as a food-producing animal as soon as it has an equine passport<br>○ I apply for an "emergency transponder" and treat the foal with medications approved for food-producing horses<br>○ I am unable to treat the foal and put it to sleep<br>○ Other: [ ]<br><br>If you select "Other", you can explain in the comment box.

F 49

**Stellen Sie Anwendungs- und Abgabebelege für Schlachtequiden aus? ***<br><br>Bitte wählen Sie nur eine der folgenden Antworten aus:<br>○ Ja, immer<br>○ Ja, manchmal<br>○ Ja, nur wenn notwendig<br>○ Ja, selten<br>○ Nie	**Do you issue a drug application and dispersion form (‘AuA-Beleg’) for slaughter equines? ***<br><br>Please select only one of the following responses:<br>○ Yes, always<br>○ Yes, sometimes<br>○ Yes, only when necessary<br>○ Yes, rarely<br>○ Never

F 50

**Für welche Medikamente stellen Sie AuA-Belege aus? ***<br><br>Diese Frage wird nur angezeigt, wenn folgende Bedingungen erfüllt sind:<br>Antwort war 'Ja, selten' oder 'Ja, nur wenn notwendig' oder 'Ja, manchmal' oder 'Ja, immer' bei Frage '49'.<br>Bitte wählen Sie nur eine der folgenden Antworten aus:<br>○ Medikamente mit Wartezeit<br>○ Antibiotika<br>○ Alle Medikamente<br>○ Alle Medikamente die nicht in den Pferdepass eingetragen werden<br>○ Sonstiges: [ ]<br><br>Falls Sie "Sonstiges" wählen, können Sie dies im Kommentarfeld erläutern.	**For which medications do you issue drug application and dispersion forms (‘AuA-Belege’)? ***<br><br>This question is only displayed if the following conditions are met:<br>Answer was 'Yes, rarely' or 'Yes, only when necessary' or 'Yes, sometimes' or 'Yes, always' for question 49.<br>Please select only one of the following answers:<br>○ Medications with a waiting period<br>○ Antibiotics<br>○ All medications<br>○ All medications that are not entered in the horse passport<br>○ Other: [ ]<br><br>If you select "Other", you can explain this in the comment field.

F 51

**Wann erhalten die Pferdehalter*innen die AuA-Belege? ***<br><br>Diese Frage wird nur angezeigt, wenn folgende Bedingungen erfüllt sind:<br>Antwort war 'Ja, selten' oder 'Ja, nur wenn notwendig' oder 'Ja, manchmal' oder 'Ja, immer' bei Frage '49'.<br>Bitte wählen Sie nur eine der folgenden Antworten aus:<br>o Unverzüglich bei Anwendung und Abgabe<br>o Meistens unverzüglich<br>o In der Regel später, z.B. bei Ausstellung der Rechnung<br>o Der/die Pferdebesitzer*in erhält den AuA-Beleg<br>o Sonstiges: ____<br><br>Falls Sie "Sonstiges" wählen, können Sie dies im Kommentarfeld erläutern.	**When does the equine keeper receive the drug administration and dispersion form (‘AuA-Beleg’)? ***<br><br>This question is only displayed if the following conditions are met:<br>Answer was 'Yes, rarely' or 'Yes, only when necessary' or 'Yes, sometimes' or 'Yes, always' for question 49.<br>Please select only one of the following responses:<br>o Immediately upon application and dispensing<br>o Usually immediately<br>o Usually later, e.g., when the invoice is issued<br>o The horse owner receives the drug application and dispersion form<br>o Other: ____<br><br>If you select "Other", you can explain this in the comment field.

F 52

**Kennen Sie die Vorgaben der sog. „Positivliste“ (VO (EG) 1950/2006) für Pferde? ***<br><br>Bitte wählen Sie die zutreffende Antwort aus:<br>o Sehr gut<br>o Gut<br>o Mäßig<br>o Schlecht<br>o Gar nicht	**How well do you know the regulations of the ‘positive list’ (Reg. (EC) No. 1950/2006)? ***<br><br>Please select the appropriate answer:<br>o Very well<br>o Well<br>o Moderately<br>o Poorly<br>o Not at all

F 53

**Wissen Sie, welche Wartezeit bei Anwendung eines Stoffes der Positivliste beachtet werden muss? ***<br><br>Bitte wählen Sie nur eine der folgenden Antworten aus:<br>o Ja<br>o Nein	**Do you know which withdrawal period must be observed when using a substance from the positive list? ***<br><br>Please select only one of the following answers:<br>o Yes<br>o No

F 54

Wie hoch ist die Wartezeit für Stoffe der Positivliste? *	What is the withdrawal period for substances on the positive list? *
Diese Frage wird nur angezeigt, wenn folgende Bedingungen erfüllt sind:<br>Antwort war 'Ja' bei Frage '53'.<br>Bitte geben Sie Ihre Antwort hier ein:	This question is only displayed if the following conditions are met:<br>Answer was 'Yes' to question 53.<br>Please enter your answer here:

F 55

Passen Sie die Wartezeit von Tierarzneimitteln bei Dosiserhöhung an?	Do you adjust the withdrawal period of veterinary medicines when the dose is increased?
Bitte wählen Sie nur eine der folgenden Antworten aus:<br>○ Ja<br>○ Nein	Please select only one of the following answers:<br>○ Yes<br>○ No

F 56

Passen Sie die Wartezeit von Stoffen der Positivliste bei Dosiserhöhung an?	Do you adjust the withdrawal period of substances from the positive list when the dosage is increased?
Bitte wählen Sie nur eine der folgenden Antworten aus:<br>○ Ja<br>○ Nein	Please select only one of the following answers:<br>○ Yes<br>○ No

F 57

Wissen Sie, wann eine Umwidmung bei einem Schlachtpferd erfolgen darf? *	Do you know when a reallocation[1] may occur in a horse for slaughter? *
Bitte wählen Sie nur eine der folgenden Antworten aus:<br>○ Ja<br>○ Nein	Please select only one of the following answers:<br>○ Yes<br>○ No

F 58

Wie erfolgt eine Umwidmung und was muss beachtet werden? *	How does a reallocation take place and what must be taken into account? *
Diese Frage wird nur angezeigt, wenn folgende Bedingungen erfüllt sind:<br>Antwort war 'Ja' bei Frage '57'.<br>Bitte geben Sie Ihre Antwort hier ein:	This question is only displayed if the following conditions are met:<br>Answer was 'Yes' to question 57.<br>Please enter your answer here:

[1] Usage that differs from the original drug registration, for example for a different indication.

F 59

**Wie oft im Monat erfolgt bei Ihnen durchschnittlich eine Umwidmung von Arzneimitteln bei Schlachtequiden? *** <br>Bitte geben Sie Ihre Antwort hier ein: [ ]	**On average, how many times a month do you have a reallocation of drugs in slaughter equines? *** <br>Please enter your answer here: [ ]

F 60

**Wissen Sie welche Wartezeit bei einer Umwidmung beachtet werden muss? *** <br>Bitte wählen Sie nur eine der folgenden Antworten aus: o Ja o Nein	**Do you know what withdrawal period must be observed for the use of a reallocated drug? *** <br>Please select only one of the following answers: o Yes o No

F 61

**Welche Wartezeit muss bei Umwidmung von Arzneimitteln beachtet werden? *** <br>Diese Frage wird nur angezeigt, wenn folgende Bedingungen erfüllt sind: Antwort war 'Ja' bei Frage '60'. Bitte geben Sie Ihre Antwort hier ein: [ ]	**What is the withdrawal period to be observed for the use of a reallocated drug? *** <br>This question is only displayed if the following conditions are met: Answer was 'Yes' to question 60. Please enter your answer here: [ ]

F 62

**Wie empfinden Sie den Dokumentationsaufwand bei Lebensmittel liefernden Equiden? *** <br>Bitte wählen Sie die zutreffende Antwort aus: o Groß o Eher groß o Mäßig o Eher gering o gering	**How do you perceive the overall documentation effort for equines destined for slaughter? *** <br>Please select the answer that applies: o Large o Rather large o Moderate o Rather small o Low

F 63

**Wie empfinden Sie die Vorgaben zur Anwendung von Arzneimitteln aus der Positivliste? *** <br>Bitte wählen Sie die zutreffende Antwort aus: o Kompliziert o Eher kompliziert o Weder kompliziert noch einfach o Eher einfach o einfach	**How do you perceive the complexity of the regulation regarding the ‘positive list’ (Reg. (EC) No. 1950/2006)? *** <br>Please select the applicable answer: o Complicated o Rather complicated o Neither complicated nor simple o Rather simple o Simple

F 64

Würden Sie einen vereinheitlichten Pferdepass unabhängig vom Zuchtverband als Vereinfachung empfinden?	Would you consider a uniformly structured equine passport regardless of the breeding association that issue them, to be a simplification?
Bitte wählen Sie nur eine der folgenden Antworten aus:<br>o Ja<br>o Nein	Please select only one of the following answers:<br>o Yes<br>o No

F 65

Würden Sie sich mehr Fortbildungen zu Arzneimitteldokumentationspflichten, insbesondere bei Lebensmittel liefernden Tieren, wünschen?	Would you like more opportunities for advanced training regarding regulations of drug administration documentations, specifically for food-producing animals?
Bitte wählen Sie nur eine der folgenden Antworten aus:<br>o Ja<br>o Nein	Please select only one of the following responses:<br>o Yes<br>o No

Feedback und Anmerkungen / Feedback and comments

F 66

Falls Sie Anmerkungen zum Fragebogen oder Ihren Antworten haben, können Sie diese in das Textfeld eintragen.<br>Bitte geben Sie Ihre Antwort hier ein:<br>[ ]<br>Vielen Dank für Ihre Teilnahme!	If you have any comments about the questionnaire or your answers, you can enter them in the text box.<br>Please enter your answer here:<br>[ ]<br>Thank you for your participation!

Display after Submission

Postanschrift:<br>Freien Universität Berlin<br>Fachbereich Veterinärmedizin<br>Institut für Lebensmittelsicherheit und –hygiene<br>AG Fleischhygiene<br>Königsweg 67, Gebäude 21/22<br>14163 Berlin<br><br>E-Mail:<br>schneides91@zedat.fu-berlin.de<br><br>Übermittlung Ihres ausgefüllten Fragebogens: Vielen Dank für die Beantwortung des Fragebogens.	Postal address:<br>Freie Universität Berlin<br>Department of Veterinary Medicine<br>Institute for Food Safety and Hygiene<br>WG Meat Hygiene<br>Königsweg 67, Building 21/22<br>14163 Berlin<br><br>eEmail:<br>schneides91@zedat.fu-berlin.de<br><br>Transmission of your completed questionnaire: Thank you very much for answering the questionnaire.

* Pflichtfrage / Mandatory question

F17 essential question; only questionnaires that had at least 17 answered questions were included.
