# Supplementary material for: Verification of documentation plausibility in equine passports–drug documentation for geldings in comparison to self-reported veterinarian drug usage for equine castrations in Germany
Source: PLoS One. 2023 Oct 18;18(10):e0292969. doi: 10.1371/journal.pone.0292969 (PMC10584153; doi:10.1371/journal.pone.0292969)
Supplement: S2 File — (PDF) [file pone.0292969.s008.pdf]

S2 File – Questionnaire for equine clinics

Praxis und Lehrmeinung bezüglich der Kastration von Equiden	Practice and doctrine regarding castration of equines.
Vielen Dank für Ihr Interesse und Ihre Unterstützung für das Forschungsvorhaben „Arzneimittelanwendung und Dokumentation bei Equiden“.<br><br>Mein Name ist Shary Schneider, ich bin Tierärztin und schreibe eine Doktorarbeit an der Freien Universität Berlin am Fachbereich Veterinärmedizin, Institut für Lebensmittelsicherheit und –hygiene.<br><br>Im Rahmen meiner Doktorarbeit befrage ich Tierbesitzer*innen, Stallbetreiber*innen und Tierärzte / Tierärztinnen zu verschiedenen Punkten der Arzneimittelanwendung bei Pferden und Eseln, sowie Pferdekliniken und Institute für Pharmakologie der deutschen veterinärmedizinischen Hochschulen.<br><br>Ziel dieser Befragung ist es, den Stand der aktuell bei Equiden praktizierten Tiermedizin abzubilden. So sollen langfristig Ansatzpunkte zur Verbesserung der Arzneimittelsicherheit bei Pferden und Eseln gefunden werden.<br><br>Der nachfolgende Fragebogen für Pferdekliniken und Pferdetierärzte / Pferdetierärztinnen umfasst Fragen zur Lehrmeinung und gelebten Praxis bezüglich Arzneimittelanwendung und Arzneimitteldokumentation bei Pferden und Eseln während/nach einer Kastration.<br><br>Die Dauer der Befragung beträgt ca. 15 Minuten.<br><br>Sämtliche Daten werden nach den Richtlinien der DSGVO (Datenschutz-Grundverordnung) streng vertraulich behandelt. Eine personenbezogene Darstellung, sowie die Weitergabe an Dritte sind grundsätzlich ausgeschlossen. Dieses Forschungsprojekt steht in keinerlei Verbindung zur behördlichen Überwachung.<br><br>Vielen Dank, dass Sie sich Zeit für die Teilnahme an dieser Studie nehmen!<br><br>In dieser Umfrage sind 26 Fragen enthalten.	Thank you for your interest and support for the research project "Drug use and documentation in equines".<br><br>My name is Shary Schneider, I am a veterinarian and I am writing a doctoral thesis at the Free University of Berlin, School of Veterinary Medicine, Institute of Food Safety and Hygiene.<br><br>As part of my doctoral thesis, I am interviewing animal owners, stable operators and veterinarians on various aspects of drug use in horses and donkeys, as well as independent equine clinics and equine clinics at German veterinary universities.<br><br>The aim of this survey is to map the state of veterinary medicine currently practiced in equines and finding starting points for long term improvement for drug safety in horses and donkeys.<br><br>The following questionnaire for equine clinics and equine veterinarians includes questions on the doctrine and practice regarding drug use and drug documentation in horses and donkeys during/after castration.<br><br>The duration of the survey is approximately 15 minutes.<br><br>All data will be treated strictly confidential according to the guidelines of the DSGVO (Data Protection Regulation). A personal representation, as well as the passing on to third parties are fundamentally excluded. This research project is in no way connected to official monitoring.<br><br>Thank you for taking the time to participate in this study!<br><br>There are 26 questions in this survey.

Allgemeine Frage / General questions

F1

Kastrieren Sie Hengste?*	Do you castrate stallions?*
Bitte wählen Sie nur eine der folgenden Antworten aus:<br><br>○ Ja, nur Pferdehengste<br>○ Ja, nur Eselhengste<br>○ Ja, Pferde- und Eselhengste<br>○ Nein	Please select only one of the following answers:<br><br>○ Yes, only horse stallions<br>○ Yes, only donkey stallions<br>○ Yes, horse and donkey stallions<br>○ No

Pferdehengste / Horse stallions

F2

Führen Sie die Kastration eines Hengstes stehend oder abgelegt durch? *	Do you perform castration of stallions standing or laid down? *
Beantworten Sie diese Frage nur, wenn folgende Bedingungen erfüllt sind:<br>Antwort war 'Ja, Pferde- und Eselhengste' *oder* 'Ja, nur Pferdehengste' bei Frage '1'<br><br>Bitte wählen Sie nur eine der folgenden Antworten aus:<br><br>○ Stehend<br>○ Abgelegt<br>○ Sowohl stehend als auch abgelegt	Answer this question only if the following conditions are met:<br>Answer was 'Yes, horse and donkey stallions' *or* 'Yes, horse stallions only' for question '1'.<br><br>Please select only one of the following answers:<br><br>○ Standing<br>○ Laid down<br>○ Both standing and laid down

F3

Welche Standardmedikation benutzen Sie zur Sedation bzw. Narkose eines Hengstes zur Kastration?*	What standard medication do you use to sedate or anesthetize a stallion for castration?*.
Beantworten Sie diese Frage nur, wenn folgende Bedingungen erfüllt sind:<br>Antwort war 'Abgelegt' *oder* 'Stehend' bei Frage '2'<br><br>Bitte geben Sie Ihre Antwort hier ein:	Answer this question only if the following conditions are met:<br>Answer was 'Laid down' *or* 'Standing' for question '2'.<br><br>Please enter your answer here:

F4

Welche Standardmedikation benutzen Sie zur Sedation bzw. Narkose eines Hengstes zur Kastration? *	What standard medication do you use to sedate or anesthetize a stallion for castration?*
Beantworten Sie diese Frage nur, wenn folgende Bedingungen erfüllt sind:<br>Antwort war 'Sowohl stehend als auch abgelegt' bei Frage '2'<br><br>Bitte geben Sie Ihre Antwort hier ein:	Answer this question only if the following conditions are met:<br>Answer was 'Both standing and laid down' for question '2'.<br><br>Please enter your answer here:

F5

Welche Medikation verwenden Sie zur Analgesie während/nach der Kastration bei einem Hengst? *	What medication do you use for analgesia during/after castration in a stallion? *
Beantworten Sie diese Frage nur, wenn folgende Bedingungen erfüllt sind:<br>Antwort war 'Ja, nur Pferdehengste' *oder* 'Ja, Pferde- und Eselhengste' bei Frage '1'<br><br>Bitte geben Sie Ihre Antwort hier ein:	Answer this question only if the following conditions are met:<br>Answer was 'Yes, horse stallions only' *or* 'Yes, horse and donkey stallions' for question '1'.<br><br>Please enter your answer here:

F6

Unterscheidet sich die Sedation / Narkose für eine Kastration je nach Schlachtstatus des Pferdes? *	Does sedation/anesthesia for castration differ depending on the slaughter status of the horse? *
Beantworten Sie diese Frage nur, wenn folgende Bedingungen erfüllt sind:<br>Antwort war 'Ja, nur Pferdehengste' *oder* 'Ja, Pferde- und Eselhengste' bei Frage '1'<br><br>Bitte wählen Sie nur eine der folgenden Antworten aus:<br><br>○ Ja<br>○ Nein	Answer this question only if the following conditions are met:<br>Answer was 'Yes, horse stallions only' *or* 'Yes, horse and donkey stallions' for question '1'.<br><br>Please select only one of the following answers:<br><br>○ Yes<br>○ No

F7

Inwiefern unterscheidet sich die Sedation / Narkose eines Schlachtpferdes?*	In what way is the sedation / anesthesia of a slaughter horse different?*.

Beantworten Sie diese Frage nur, wenn folgende Bedingungen erfüllt sind:<br>Antwort war 'Ja' bei Frage '6'<br><br>Bitte geben Sie Ihre Antwort hier ein:	Answer this question only if the following conditions are met:<br>Answer was 'Yes' to question '6'.<br><br>Please enter your answer here:

F8

**Unterscheidet sich die Medikation zur Analgesie während bzw. nach Kastration je nach Schlachtstatus des Pferdes? ***<br><br>Beantworten Sie diese Frage nur, wenn folgende Bedingungen erfüllt sind:<br>Antwort war 'Ja, nur Pferdehengste' *oder* 'Ja, Pferde- und Eselhengste' bei Frage '1'<br><br>Bitte wählen Sie nur eine der folgenden Antworten aus:<br><br>o Ja<br>o Nein	**Does analgesia medication during or after castration differ depending on the slaughter status of the horse? ***<br><br>Answer this question only if the following conditions are met:<br>Answer was 'Yes, horse stallions only' *or* 'Yes, horse and donkey stallions' for question '1'.<br><br>Please select only one of the following answers:<br><br>o Yes<br>o No

F9

**Inwiefern unterscheidet sich die analgetische Medikation eines Schlachtpferdes? ***<br><br>Beantworten Sie diese Frage nur, wenn folgende Bedingungen erfüllt sind:<br>Antwort war 'Ja' bei Frage '8'<br>Bitte geben Sie Ihre Antwort hier ein:	**How does analgesic medication of a slaughter horse differ? ***<br><br>Answer this question only if the following conditions are met:<br>Answer was 'Yes' to question '8'.<br>Please enter your answer here:

F10

**Unterscheidet sich die Dokumentation der angewendeten Medikamente je nach Schlachtstatus des Pferdes? ***<br><br>Beantworten Sie diese Frage nur, wenn folgende Bedingungen erfüllt sind:<br>Antwort war 'Ja, nur Pferdehengste' *oder* 'Ja, Pferde- und Eselhengste' bei Frage '1'<br>Bitte wählen Sie nur eine der folgenden Antworten aus:<br><br>o Ja<br>o Nein	**Does the documentation of medications used differ depending on the slaughter status of the horse? ***<br>Answer this question only if the following conditions are met:<br>Answer was 'Yes, horse stallions only' *or* 'Yes, horse and donkey stallions' for question '1'.<br>Please select only one of the following answers:<br><br>o Yes<br>o No

F11

**Wie unterscheidet sich die durchgeführte Dokumentation? ***<br><br>Beantworten Sie diese Frage nur, wenn folgende Bedingungen erfüllt sind:<br>Antwort war 'Ja' bei Frage '10'<br>Bitte geben Sie Ihre Antwort hier ein:<br>[ ]	**How does the documentation required differ? ***<br><br>Answer this question only if the following conditions are met:<br>Answer was 'Yes' to question '10'.<br>Please enter your answer here:<br>[ ]

Eselhengste / Donkey stallions

F12

**Führen Sie die Kastration eines Eselhengstes stehend oder abgelegt durch? ***<br><br>Beantworten Sie diese Frage nur, wenn folgende Bedingungen erfüllt sind:<br>Antwort war 'Ja, nur Eselhengste' *oder* 'Ja, Pferde- und Eselhengste' bei Frage '1'<br>Bitte wählen Sie nur eine der folgenden Antworten aus:<br><br>o Stehend<br>o Abgelegt<br>o Sowohl stehend als auch abgelegt	**Do you perform castration of a donkey stallion standing up or laying down? ***<br><br>Answer this question only if the following conditions are met:<br>Answer was 'Yes, donkey stallions only' or 'Yes, horse and donkey stallions' for question '1'.<br>Please select only one of the following answers:<br><br>o Standing<br>o Laid down<br>o Both standing and laid down

F13

**Welche Standardmedikation benutzen Sie zur Sedation bzw. Narkose eines Eselhengstes zur Kastration? ***<br><br>Beantworten Sie diese Frage nur, wenn folgende Bedingungen erfüllt sind:<br>Antwort war 'Abgelegt' *oder* 'Stehend' bei Frage '12'<br>Bitte geben Sie Ihre Antwort hier ein:<br>[ ]	**What standard medication do you use to sedate or anesthetize a donkey stallion for castration? ***<br><br>Answer this question only if the following conditions are met:<br>Answer was 'Laid Down' *or* 'Standing' for question '12'.<br>Please enter your answer here:<br>[ ]

F14

**Welche Standardmedikation benutzen Sie zur Sedation bzw. Narkose eines Eselhengstes zur Kastration? ***<br><br>Beantworten Sie diese Frage nur, wenn folgende Bedingungen erfüllt sind:	**What standard medication do you use to sedate or anesthetize a donkey stallion for castration? ***<br><br>Answer this question only if the following conditions are met:

Antwort war 'Sowohl stehend als auch abgelegt' bei Frage '12'<br>Bitte geben Sie Ihre Antwort hier ein:	Answer was 'Both standing and laying down' for question '12'.<br>Please enter your answer here:

F15

**Welche Medikation verwenden Sie zur Analgesie während/nach der Kastration bei einem Eselhengst? ***<br><br>Beantworten Sie diese Frage nur, wenn folgende Bedingungen erfüllt sind:<br>Antwort war 'Ja, nur Eselhengste' *oder* 'Ja, Pferde- und Eselhengste' bei Frage '1'<br>Bitte geben Sie Ihre Antwort hier ein:	**What medication do you use for analgesia during/after castration in a donkey stallion? ***<br><br>Answer this question only if the following conditions are met:<br>Answer was 'Yes, donkey stallions only' *or* 'Yes, horse and donkey stallions' for question '1'.<br>Please enter your answer here:

F16

**Unterscheidet sich die Sedation / Narkose für eine Kastration je nach Schlachtstatus des Esels? ***<br><br>Beantworten Sie diese Frage nur, wenn folgende Bedingungen erfüllt sind:<br>Antwort war 'Ja, nur Eselhengste' *oder* 'Ja, Pferde- und Eselhengste' bei Frage '1'<br>Bitte wählen Sie nur eine der folgenden Antworten aus:<br><br>o Ja<br>o Nein	**Does sedation/anesthesia for castration differ depending on the slaughter status of the donkey? ***<br><br>Answer this question only if the following conditions are met:<br>Answer was 'Yes, donkey stallions only' *or* 'Yes, horse and donkey stallions' for question '1'.<br>Please select only one of the following answers:<br><br>o Yes<br>o No

F17

**Inwiefern unterscheidet sich die Sedation / Narkose eines Schlachtesels? ***<br><br>Beantworten Sie diese Frage nur, wenn folgende Bedingungen erfüllt sind:<br>Antwort war 'Ja' bei Frage '16'<br>Bitte geben Sie Ihre Antwort hier ein:	**How is sedation/anesthesia of a slaughter donkey different? ***<br><br>Answer this question only if the following conditions are met:<br>Answer was 'Yes' to question '16'.<br>Please enter your answer here:

F18

Unterscheidet sich die Medikation zur Analgesie während bzw. nach Kastration je nach Schlachtstatus des Esels? *	Does analgesia medication during or after castration differ depending on the slaughter status of the donkey? *
Beantworten Sie diese Frage nur, wenn folgende Bedingungen erfüllt sind:<br>Antwort war 'Ja, nur Eselhengste' *oder* 'Ja, Pferde- und Eselhengste' bei Frage '1'<br>Bitte wählen Sie nur eine der folgenden Antworten aus:<br><br>o Ja<br>o Nein	Answer this question only if the following conditions are met:<br>Answer was 'Yes, donkey stallions only' *or* 'Yes, horse and donkey stallions' for question '1'.<br>Please select only one of the following answers:<br><br>o Yes<br>o No

F19

Inwiefern unterscheidet sich die analgetische Medikation eines Schlachtesels? *	How does analgesic medication of a slaughter donkey differ? *
Beantworten Sie diese Frage nur, wenn folgende Bedingungen erfüllt sind:<br>Antwort war 'Ja' bei Frage '18'<br>Bitte geben Sie Ihre Antwort hier ein:	Answer this question only if the following conditions are met:<br>Answer was 'Yes' to question '18'.<br>Please enter your answer here:

F20

Unterscheidet sich die Dokumentation der angewendeten Medikamente je nach Schlachtstatus des Esels? *	Does the documentation of medications applied differ depending on the slaughter status of the donkey? *
Beantworten Sie diese Frage nur, wenn folgende Bedingungen erfüllt sind:<br>Antwort war 'Ja, nur Eselhengste' *oder* 'Ja, Pferde- und Eselhengste' bei Frage '1'<br>Bitte wählen Sie nur eine der folgenden Antworten aus:<br><br>o Ja<br>o Nein	Answer this question only if the following conditions are met:<br>Answer was 'Yes, donkey stallions only' *or* 'Yes, horse and donkey stallions' for question '1'.<br>Please select only one of the following answers:<br><br>o Yes<br>o No

F21

Wie unterscheidet sich die durchgeführte Dokumentation? *	How does the documentation required differ? *
Beantworten Sie diese Frage nur, wenn folgende Bedingungen erfüllt sind:<br>Antwort war 'Ja' bei Frage '20'	Answer this question only if the following conditions are met:<br>Answer was 'Yes' to question '20'.

Bitte geben Sie Ihre Antwort hier ein:	Please enter your answer here:

Medikation / Medication

F22

**Wie häufig weichen Sie von der von Ihnen bei einer Kastration angewendeten Standardmedikation ab?***<br><br>Beantworten Sie diese Frage nur, wenn folgende Bedingungen erfüllt sind:<br>Antwort war 'Ja, nur Pferdehengste' *oder* 'Ja, nur Eselhengste' *oder* 'Ja, Pferde- und Eselhengste' bei Frage '1'<br>Bitte wählen Sie nur eine der folgenden Antworten aus:<br><br>○ <5%<br>○ 5% bis <10%<br>○ 10% bis <25%<br>○ 25% bis ≤50%	**How often do you deviate from the standard medication you use during a castration?***<br><br>Answer this question only if the following conditions are met:<br>Answer was 'Yes, horse stallions only' *or* 'Yes, donkey stallions only' *or* 'Yes, horse and donkey stallions' for question '1'.<br>Please select only one of the following responses:<br><br>○ <5%<br>○ 5% to <10%<br>○ 10% to <25%<br>○ 25% to ≤50%

F23

**Was sind die häufigsten Gründe für ein Abweichen von der Standardmedikation bei Kastrationen?***<br><br>Beantworten Sie diese Frage nur, wenn folgende Bedingungen erfüllt sind:<br>Antwort war 'Ja, nur Pferdehengste' *oder* 'Ja, nur Eselhengste' *oder* 'Ja, Pferde- und Eselhengste' bei Frage '1'<br>Bitte geben Sie Ihre Antwort hier ein:<br>Bitte nennen Sie maximal drei Gründe.	**What are the most common reasons for deviating from the standard medication for neutering?*.**<br><br>Answer this question only if the following conditions are met:<br>Answer was 'Yes, horse stallions only' *or* 'Yes, donkey stallions only' *or* 'Yes, horse and donkey stallions' for question '1'.<br>Please enter your answer here:<br>Please give a maximum of three reasons.

F24

**Ist die Behandlung von Narkosezwischenfällen bei einem Schlachtequiden erschwert?***<br><br>Beantworten Sie diese Frage nur, wenn folgende Bedingungen erfüllt sind:<br>Antwort war 'Ja, nur Pferdehengste' *oder* 'Ja, nur Eselhengste' *oder* 'Ja, Pferde- und Eselhengste' bei Frage '1'<br>Bitte wählen Sie nur eine der folgenden Antworten aus:	**Is the treatment of anesthetic incidents more difficult in a slaughter equines?***<br><br>Answer this question only if the following conditions are met:<br>Answer was 'Yes, horse stallions only' *or* 'Yes, donkey stallions only' *or* 'Yes, horse and donkey stallions' for question '1'.<br>Please select only one of the following answers:<br><br>○ Yes

○ Ja<br>○ Nein	○ No

F25

**Inwiefern ist die Behandlung von Narkosezwischenfällen bei Schlachtequiden erschwert? ***<br><br>Beantworten Sie diese Frage nur, wenn folgende Bedingungen erfüllt sind:<br>Antwort war 'Ja' bei Frage '24'<br>Bitte geben Sie Ihre Antwort hier ein:	**To what extent is the management of anesthetic incidents more difficult in slaughter equines? ***<br><br>Answer this question only if the following conditions are met:<br>Answer was 'Yes' to question '24'.<br>Please enter your answer here:

Feedback / Feedback

F26

**Falls Sie Anmerkungen zum Fragebogen oder Ihren Antworten haben, können Sie diese in das Freitextfeld eintragen.**<br><br>Bitte geben Sie Ihre Antwort hier ein:	**If you have any comments about the questionnaire or your answers, you can enter them in the text box.**<br><br>Please enter your answer here:

Display after Submission

Vielen Dank für Ihre Teilnahme!	Thank you for your participation!
Postanschrift:<br><br>Freien Universität Berlin<br>Fachbereich Veterinärmedizin<br>Institut für Lebensmittelsicherheit und –hygiene<br>AG Fleischhygiene<br>Königsweg 67, Gebäude 21/22<br>14163 Berlin<br><br>E-Mail:<br>schneides91@zedat.fu-berlin.de<br><br>Übermittlung Ihres ausgefüllten Fragebogens:<br>Vielen Dank für die Beantwortung des Fragebogens.	Postal address:<br><br>Freie Universität Berlin<br>Department of Veterinary Medicine<br>Institute for Food Safety and Hygiene<br>WG Meat Hygiene Königsweg 67, Building 21/22<br>14163 Berlin<br><br>eEmail: schneides91@zedat.fu-berlin.de<br><br>Transmission of your completed questionnaire:<br>Thank you very much for answering the questionnaire.
