# Supplementary material for: Verification of documentation plausibility in equine passports–drug documentation for geldings in comparison to self-reported veterinarian drug usage for equine castrations in Germany
Source: PLoS One. 2023 Oct 18;18(10):e0292969. doi: 10.1371/journal.pone.0292969 (PMC10584153; doi:10.1371/journal.pone.0292969)
Supplement: S3 File — (PDF) [file pone.0292969.s009.pdf]

Equine passport inspection form:

□ Horse □ Donkey

□ Male □ Female □ Castrated

Year of birth: ___________________

Is the castration documented in the equine passport?

□ Yes Date of documentation: __________________

□ No

Slaughter status:

□ Slaughter equine □ Companion equine

Date of documentation of slaughter status: __________________

□ Documentation incomplete

Entries in Annex for drug documentation (section IX part III) of the equine passport:

□ Yes □ No

If yes: what drugs are listed?

____

____

____
