# Supplementary material for: Verification of documentation plausibility in equine passports–drug documentation for geldings in comparison to self-reported veterinarian drug usage for equine castrations in Germany
Source: PLoS One. 2023 Oct 18;18(10):e0292969. doi: 10.1371/journal.pone.0292969 (PMC10584153; doi:10.1371/journal.pone.0292969)
Supplement: S4 File — (DOCX) [file pone.0292969.s010.docx]

STROBE Statement—checklist of items that should be included in reports of observational studies

|  | Item No. | Recommendation | Page  No. | | Relevant text from manuscript | |
| --- | --- | --- | --- | --- | --- | --- |
| **Title and abstract** | 1 | (*a*) Indicate the study’s design with a commonly used term in the title or the abstract | 1 | Self-reported | | |
|  |  | (*b*) Provide in the abstract an informative and balanced summary of what was done and what was found | 2 | This study compared the results of two sub-studies. In sub-study 1, 116 veterinarians and nine equine clinics in Germany were surveyed about the methods and drugs used for castration of equine stallions. In sub-study 2, the documentational findings of 195 equine passports, belonging to 194 horses and one donkey, were analyzed.  Regarding sub-study 1, the most commonly used method for castration was reported as ‘laid down’. Drug combinations entailing at least one drug from the ‘positive list’ were used by 86.7 % (91/105) of veterinarians castrating horse stallions ‘laid down’ and by 61.0 % (36/56) of veterinarians utilizing this method on donkey stallions. Regarding sub-study 2, drug documentation was verified in the passports of 4.6 % (9/195) of all equines and in just 12.0 % (3/25) of those belonging to slaughter equine geldings. Anesthetics from the ‘positive list’ were documented in 4.0 % (1/25) of equine passports belonging to slaughter geldings. | | |
| Introduction | | | | | |  |
| Background/rationale | 2 | Explain the scientific background and rationale for the investigation being reported | 3 | The equine passport was introduced in the European Union (EU) through Regulation (EC) No 504/2008 with the goal to implement an identification document for horses, donkeys, zebras, and hybrids thereof and to allow differentiation between slaughter and companion equines [1]. In 2015, the regulation was lifted through the implementation of the Implementing Regulation (EC) 2015/262, which in return was replaced by Implementing Regulation (EU) 2021/963 in July 2021 [2, 3]. To ensure adequate treatment options and access to medications of critical importance for slaughter equines, the so-called ‘positive list for equines’ (Regulation (EC) No 1950/2006), was compiled [4]. The ‘positive list’ is a directory with medications that are approved for use in slaughter equines under specific conditions in addition to medications approved for other livestock, as set in Table 1 of Regulation (EU) No 37/2010 [4, 5]. There are different regulations in place for the usage of medications on the ‘positive list’ than for other livestock-approved medications. For all medication listed in the ‘positive list’, the withdrawal period is six months, and each usage has to be documented in the equine passport. Another particularity regarding equines in the European legislation is the differentiation between slaughter equines (meat or other products from these animals can enter the human food chain) and companion equines (not allowed to be slaughtered for entry to the human food chain). For the treatment of companion equines, all medications that are allowed to be used in animals can be administered. However, if an equine is intended for slaughter to obtain meat for human consumption, certain medications are banned from use (usage of these medications is prohibited in livestock) or can only be used in compliance with particular regulations (medications on the ‘positive list’) [4, 5].  Because of the frequent changes in the regulations regarding the equine passport and amendments to the ‘positive list’, as well as the differentiation between slaughter and companion equines, the usage of medication in equines presents the veterinarians in Germany and the European Union with difficulties not faced in the treatment of other livestock or companion animals [1-4]. | | |
| Objectives | 3 | State specific objectives, including any prespecified hypotheses | 4 | This leads to the assumption that the documentation of medications in equine passports is likely insufficient and incomplete. To verify this thesis, we conducted a study with three goals. The first one was to generate an overview about the commonly used medications for the different ways to castrate horse and donkey stallions by veterinarians in Germany. The second goal was to determine how often medications are documented in the equine passport, especially anesthetics used during castration. Lastly, the goal was to determine if the frequency of usage of certain medication correlates with the frequency of documentation of the respective medication in the equine passports. | | |
| Methods | | | | | |  |
| Study design | 4 | Present key elements of study design early in the paper | 4 | Data collection was performed via two different ways: 1) an online survey (active between 19^th^ October and 15^th^ December 2021) for different target groups and 2) through inspection of equine passports of individual animals at the clinic for horses of Freie Universität Berlin and in private stables (1^st^ June to 31^st^ December 2021). | | |
| Setting | 5 | Describe the setting, locations, and relevant dates, including periods of recruitment, exposure, follow-up, and data collection | 4-7 | | |  |
| Participants | 6 | (*a*) *Cohort study*—Give the eligibility criteria, and the sources and methods of selection of participants. Describe methods of follow-up  *Case-control study*—Give the eligibility criteria, and the sources and methods of case ascertainment and control selection. Give the rationale for the choice of cases and controls  *Cross-sectional study*—Give the eligibility criteria, and the sources and methods of selection of participants | 6 | “For responses to be included in the study, a minimum of 17 questions had to be answered, and the veterinarians had to specify which method for castrating equine stallions was applied.” | | |
|  |  | (*b*) *Cohort study*—For matched studies, give matching criteria and number of exposed and unexposed  *Case-control study*—For matched studies, give matching criteria and the number of controls per case |  | | |  |
| Variables | 7 | Clearly define all outcomes, exposures, predictors, potential confounders, and effect modifiers. Give diagnostic criteria, if applicable |  | Not applicable | | |
| Data sources/ measurement | 8* | For each variable of interest, give sources of data and details of methods of assessment (measurement). Describe comparability of assessment methods if there is more than one group | 6 | | |  |
| Bias | 9 | Describe any efforts to address potential sources of bias | 18 | “Since the equine passport inspections only included the passport of one donkey, the results cannot be representative of the German donkey population.”; “For both the veterinarian and clinic results, some selection bias has to be mentioned, since the study was online, and consequently, only veterinarians and clinics with internet access, which is broadly available in Germany, were included.” | | |
| Study size | 10 | Explain how the study size was arrived at | 6 | Veterinarians: “For responses to be included in the study, a minimum of 17 questions had to be answered, and the veterinarians had to specify which method for castrating equine stallions was applied.”  Clinics: “For responses to be included in the study, all displayed questions had to be answered.” | | |

Continued on next page

| Quantitative variables | 11 | Explain how quantitative variables were handled in the analyses. If applicable, describe which groupings were chosen and why |  | Not applicable | | |
| --- | --- | --- | --- | --- | --- | --- |
| Statistical methods | 12 | (*a*) Describe all statistical methods, including those used to control for confounding | 7 | “Descriptive data analysis was performed using and IBM SPSS version 28.0 for Windows (IBM® - Armonk, New York, USA). Frequency tables and figures were configured.” | | |
|  |  | (*b*) Describe any methods used to examine subgroups and interactions |  | Not applicable | | |
|  |  | (*c*) Explain how missing data were addressed |  | Not applicable | | |
|  |  | (*d*) *Cohort study*—If applicable, explain how loss to follow-up was addressed  *Case-control study*—If applicable, explain how matching of cases and controls was addressed  *Cross-sectional study*—If applicable, describe analytical methods taking account of sampling strategy |  | Not applicable | | |
|  |  | (*e*) Describe any sensitivity analyses |  | Not applicable | | |
| Results | | | | | | |
| Participants | 13* | (a) Report numbers of individuals at each stage of study—eg numbers potentially eligible, examined for eligibility, confirmed eligible, included in the study, completing follow-up, and analysed | 7 | “In the following, the results of 116 veterinarians´ and nine clinics´ questionnaires and 195 equine passports are presented.”  The study consists of only one stage. | | |
|  |  | (b) Give reasons for non-participation at each stage |  | Not applicable | | |
|  |  | (c) Consider use of a flow diagram |  | | |  |
| Descriptive data | 14* | (a) Give characteristics of study participants (eg demographic, clinical, social) and information on exposures and potential confounders | 14 | | In total, the equine passports of 195 equines were inspected, which belonged to 194 (99.5 %) horses and one female donkey (0.5 %); of all passports, 54.9 % were for males and 45.1 % for females. Of the male equines, 98 of 107 (91.6 %) were castrated. For 65 of 98 geldings (66.3 %), the castration was not documented in the equine passport. On average, the equines were born in 2009, with the oldest horse born in 1990 and the youngest in 2021. | |
|  |  | (b) Indicate number of participants with missing data for each variable of interest |  | | Not applicable | |
|  |  | (c) *Cohort study*—Summarise follow-up time (eg, average and total amount) |  | | Not applicable | |
| Outcome data | 15* | *Cohort study*—Report numbers of outcome events or summary measures over time |  | | Not applicable | |
|  |  | *Case-control study—*Report numbers in each exposure category, or summary measures of exposure |  | | Not applicable | |
|  |  | *Cross-sectional study—*Report numbers of outcome events or summary measures |  | | Not applicable | |
| Main results | 16 | (*a*) Give unadjusted estimates and, if applicable, confounder-adjusted estimates and their precision (eg, 95% confidence interval). Make clear which confounders were adjusted for and why they were included |  | | Not applicable | |
|  |  | (*b*) Report category boundaries when continuous variables were categorized |  | | Not applicable | |
|  |  | (*c*) If relevant, consider translating estimates of relative risk into absolute risk for a meaningful time period |  | | Not applicable | |

Continued on next page

| Other analyses | 17 | Report other analyses done—eg analyses of subgroups and interactions, and sensitivity analyses |  | Not applicable |
| --- | --- | --- | --- | --- |
| Discussion | | | | |
| Key results | 18 | Summarise key results with reference to study objectives | 16 | Accordingly, we expected to find documentation of administered anesthetics from the ‘positive list’ in 30 % to 87 % of the equine passports belonging to gelding horses and in 30 % to 100 % of passports belonging to gelding donkeys, that are slaughter equines. Taking into consideration the distribution of the used methods, the percentage of drug documentation findings in equine passports should be closer to 87 % and 100 % for horse and donkey geldings, respectively, than to 30 %. However, administered drugs were documented only in 12 % (3/25) of equine passports belonging to slaughter equine geldings. Most notably, only 4 % (1/25) of the equine passports belonging to slaughter equine geldings contained documentation of anesthetics from the ‘positive list’. |
| Limitations | 19 | Discuss limitations of the study, taking into account sources of potential bias or imprecision. Discuss both direction and magnitude of any potential bias | 18 | “Since the equine passport inspections only included the passport of one donkey, the results cannot be representative of the German donkey population.”; “For both the veterinarian and clinic results, some selection bias has to be mentioned, since the study was online, and consequently, only veterinarians and clinics with internet access, which is broadly available in Germany, were included.” |
| Interpretation | 20 | Give a cautious overall interpretation of results considering objectives, limitations, multiplicity of analyses, results from similar studies, and other relevant evidence | 18 | In conclusion, drug documentation in passports of equines intended for slaughter is seldom completed, leading to a potential health risk of drug residue consumption for equine meat consumers in Germany, as well as compromising the health of the individual equines. Further research is necessary to determine if missing and incomplete documentation castrations and administered drugs and in equine passports is a problem in the European Union. |
| Generalisability | 21 | Discuss the generalisability (external validity) of the study results | 18 | Acknowledging the limitations of the study, we stand by our assumption that the lack of documentation in equine passports, and so the associated risks for equine meat consumer and the equine population can be generalized for Germany. |
| Other information | |  | | |
| Funding | 22 | Give the source of funding and the role of the funders for the present study and, if applicable, for the original study on which the present article is based | Funding statement | Open Access Funding of Freie Universität Berlin; The funder had no role in the study, data collection or publication. |

*Give information separately for cases and controls in case-control studies and, if applicable, for exposed and unexposed groups in cohort and cross-sectional studies.

**Note:** An Explanation and Elaboration article discusses each checklist item and gives methodological background and published examples of transparent reporting. The STROBE checklist is best used in conjunction with this article (freely available on the Web sites of PLoS Medicine at http://www.plosmedicine.org/, Annals of Internal Medicine at http://www.annals.org/, and Epidemiology at http://www.epidem.com/). Information on the STROBE Initiative is available at www.strobe-statement.org.
